# Metagenomic and culture-dependent approaches unveil active microbial community and novel functional genes involved in arsenic mobilization and detoxification in groundwater

**DOI:** 10.1186/s12866-023-02980-0

**Published:** 2023-08-30

**Authors:** Farzana Diba, M. Nazmul Hoque, M. Shaminur Rahman, Farhana Haque, Khondaker Md. Jaminur Rahman, Md. Moniruzzaman, Mala Khan, M. Anwar Hossain, Munawar Sultana

**Affiliations:** 1 Department of Microbiology, University of Dhaka, Dhaka, 1000 Bangladesh; 2 Institute of Tissue Banking and Biomaterial Research, Atomic Energy Research Establishment, Savar, Dhaka, 1349 Bangladesh; 3 Department of Gynecology, Obstetrics and Reproductive Health, Bangabandhu Sheikh Mujibur Rahman Agricultural University, Gazipur, 1706 Bangladesh; 4 Department of Microbiology, Jashore University of Science and Technology, Jashore, 7408 Bangladesh; 5 Environmental Science Division, National Institute of Biotechnology, Savar, Dhaka, 1349 Bangladesh; 6 Bangladesh Reference Institute for Chemical Measurements (BRiCM), Dr. Qudrat-E-Khuda Road, Dhanmondi, Dhaka 1205 Bangladesh; 7 Present address: Jashore University of Science and Technology, Jashore, 7408 Bangladesh

**Keywords:** Arsenotrophic bacteria, Arsenotrophic genes, Whole-genome shotgun sequencing, AMRs, VFGs, Bioremediation

## Abstract

**Background:**

Arsenic (As) and its species are major pollutants in ecological bodied including groundwater in Bangladesh rendering serious public health concern. Bacteria with arsenotrophic genes have been found in the aquifer, converting toxic arsenite [As (III)] to less toxic arsenate [As (V)] that is easily removed using chemical and biological trappers. In this study, genomic and metagenomic approaches parallel to culture-based assay (Graphical abstract) have made it possible to decipher phylogenetic diversity of groundwater arsenotrophic microbiomes along with elucidation of their genetic determinants.

**Results:**

Seventy-two isolates were retrieved from six As-contaminated (average As concentration of 0.23 mg/L) groundwater samples from Munshiganj and Chandpur districts of Bangladesh. Twenty-three isolates harbored arsenite efflux pump (*ars*B) gene with high abundance, and ten isolates possessing arsenite oxidase (*aio*A) gene, with a wide range of minimum inhibitory concentration, MIC_As_ (2 to 32 mM), confirming their role in arsenite metabolism. There was considerable heterogeneity in species richness and microbial community structure. Microbial taxa from Proteobacteria, Firmicutes and Acidobacteria dominated these diversities. Through these combinatorial approaches, we have identified potential candidates such as, *Pseudomonas*, *Acinetobacter*, *Stenotrophomonas*, *Achromobacter*, *Paraburkholderia*, *Comamonas* and *Klebsiella* and associated functional genes (*ars*B, *acr*3, *ars*D, *ars*H, *ars*R) that could significantly contribute to arsenite detoxification, accumulation, and immobilization.

**Conclusions:**

Culture-dependent and -independent shotgun metagenomic investigation elucidated arsenotrophic microbiomes and their functions in As biogeochemical transformation. These findings laid a foundation for further large-scale researches on the arsenotrophic microbiomes and their concurrent functions in As biogeochemical transformation in As-contaminated areas of Bangladesh and beyond.

**Supplementary Information:**

The online version contains supplementary material available at 10.1186/s12866-023-02980-0.

## Background

Arsenic (As) is a highly hazardous pollutant found in the aquifers and soil that causes serious health problems globally. According to the world health organization (WHO) report 2018, at least 140 million people of 50 countries are exposed to As through arsenic-contaminated groundwater (GW) at levels more than 10 µg/L, and a majority of them live in India and Bangladesh [1]. In Bangladesh, around 95% of rural and 70% of urban populations use GW for drinking, irrigation, and household purposes, necessitating thousands of wells [2]. Arsenic levels in irrigation and drinking water are rising across Southeast Asia [3]. Bangladesh is largely located on the Bengal Basin formed by the Ganga–Brahmaputra–Meghna (GBM) river system. This sedimentary basin has been formed by deposition of large volumes of As-containing sediments that originated mainly from the Himalayas and was carried down by the mighty GBM rivers during the Pleistocene and Holocene periods. From these sediments, As is leaching into the groundwater aquifers located in the fan deposit areas and Holocene alluvium [1]. Bangladesh, the largest deltaic land in the world, is largely a low-lying floodplain with about 75% of the land being less than three meters above the sea level [1]. Tube well water is the primary source of drinking water for > 95% of the rural people of Bangladesh. Seventy-five million people of Bangladesh in 59 (out of 64) districts are chronically exposed to water having > 50 µg/L As [2, 4] which is much higher than the WHO acceptable limit of 10 µg/L [1, 3]. High risk As affected districts of Bangladesh includes Chandpur, Munshiganj, Gopalganj, Madaripur, Noakhali, Sathkhira, Cumilla, Faridpur, Shariatpur, Meherpur and Bagerhat [2]. According to a feasibility report by DPHE (Department of Public Health Engineering), about 29% of tube well out of the 4.95 million were As-contaminated (https://rb.gy/3h87n6).

Arsenic in GW exists primarily as oxy anions representing two oxidation states: [As (III)] and [As (V)] [3]. As [(III)] is 100 times more harmful [2], and more challenging to mitigate. Conversely, As (V) is more successfully eliminated than As (III) using traditional methods such as precipitation and adsorption [2]. Conventional treatments are expensive and detrimental to the environment. Bacteria strongly influence the biotransformation, detoxification, and redox transformation of arsenic. Microorganisms can cycle iron and As via redox reactions. Chemolithotrophic microorganisms obtain energy from ferrous iron and As (III). Anaerobic organisms utilize oxygen or nitrate as electron acceptors, whereas heterotrophic species use ferric iron and arsenate [5, 6]. Bacteria use toxic arsenic as an energy source for metabolic activity and survival via biosorption, intracellular bioaccumulation, and enzymatic conversion to a less toxic oxidation state [2, 7]. Since As transforming microorganisms alter the solubility, mobility, and bioavailability of As, they play important roles in the biogeochemical cycle of As [8]. This concern has prompted studies of microbial populations in aquifers undertaken with a view to identifying the bacteria responsible for As-affected of GW in Bangladesh. In groundwater, complex bacterial communities have been deciphered at the genome level, evidencing inter-organism interactions involved in ecosystem plasticity [2, 9]. Diverse bacterial communities in As-rich GW have been reported to be dominated by Firmicutes, Proteobacteria, Actinobacteria, and Cyanobacteria [2, 6, 10]. To elucidate the distribution, phylogeny and activity of the arsenotrophic bacteria in As-contaminated GW, functional molecular markers have been applied [2, 10, 11]. Arsenic-resistance (*ars*) operons encoding *ars*R, *ars*D, *ars*A, *ars*B, *ars*C, *ars*H genes are frequently observed in As-resistant microorganisms (ARMs). Most commonly reported Proteobacteria with *ars* operons are *Escherichia coli*, *Pseudomonas aeruginosa*, *Acidiphilum multivorum*, *Staphylococci*, *Bacillus subtilis*, a variety of *Yersinia* species, *Thiobacill* etc. [2, 10–12]. Arsenite-oxidizing bacteria (AOB) harboring As (III) oxidase (*aioA*) gene has been reported to detoxify As (III) to As (V) [2, 13]. Arsenite-oxidizing bacteria comprises several genera including *Pseudomonas*, *Alcaligenes*, *Thermus*, *Agrobacterium*, *Herminiimon*, *Thiomonas* and *Achromobacter* [14–16]. The *arx*-containing oxidation mechanism includes arsenite oxidation and nitrate respiration [17]. Numerous arsenate-reducing bacteria such as *S. aureus*, *Chrysiogenes arsenates*, *Geospirillum barnessi*, *B. arsenicoselenatis*, *Bacillus* spp., *Desulfitobacterium* spp., *Sulfurospirillum* spp., *Geobacter* spp., *Anaeromyxobacter* spp. and *Shewanella* spp. contain the *arr* operon, which encodes arsenate reductases (*arr*A) that enhance As release from sediment in anoxic GW through dissimilative reduction [14, 15, 17]. Both arsenite-oxidizing and arsenic-resistant bacteria can contribute to arsenic bioremediation [18, 19]. Heavy metals in different ionic states can impact microbial composition and metabolism [20]. Many of the earlier studies demonstrated that co-existence heavy metals and antibiotics contributes to the amplification of antibiotic resistance genes (ARGs) in the environmental microbiomes through diverse mechanisms and pathways (e.g., target replacement, efflux pumps, antibiotics inactivation etc.) which may ultimately transfer to the clinical settings [21]. Antimicrobial-resistant bacteria (AMR), including pathogenic strains of *E. coli*, *Salmonella*, *Legionella*, and *Pseudomonas aeruginosa*, have invaded drinking water systems and possess ARGs such as *tet*A, *sul*1, and *sul*2 [22, 23].

**Fig. 1:**
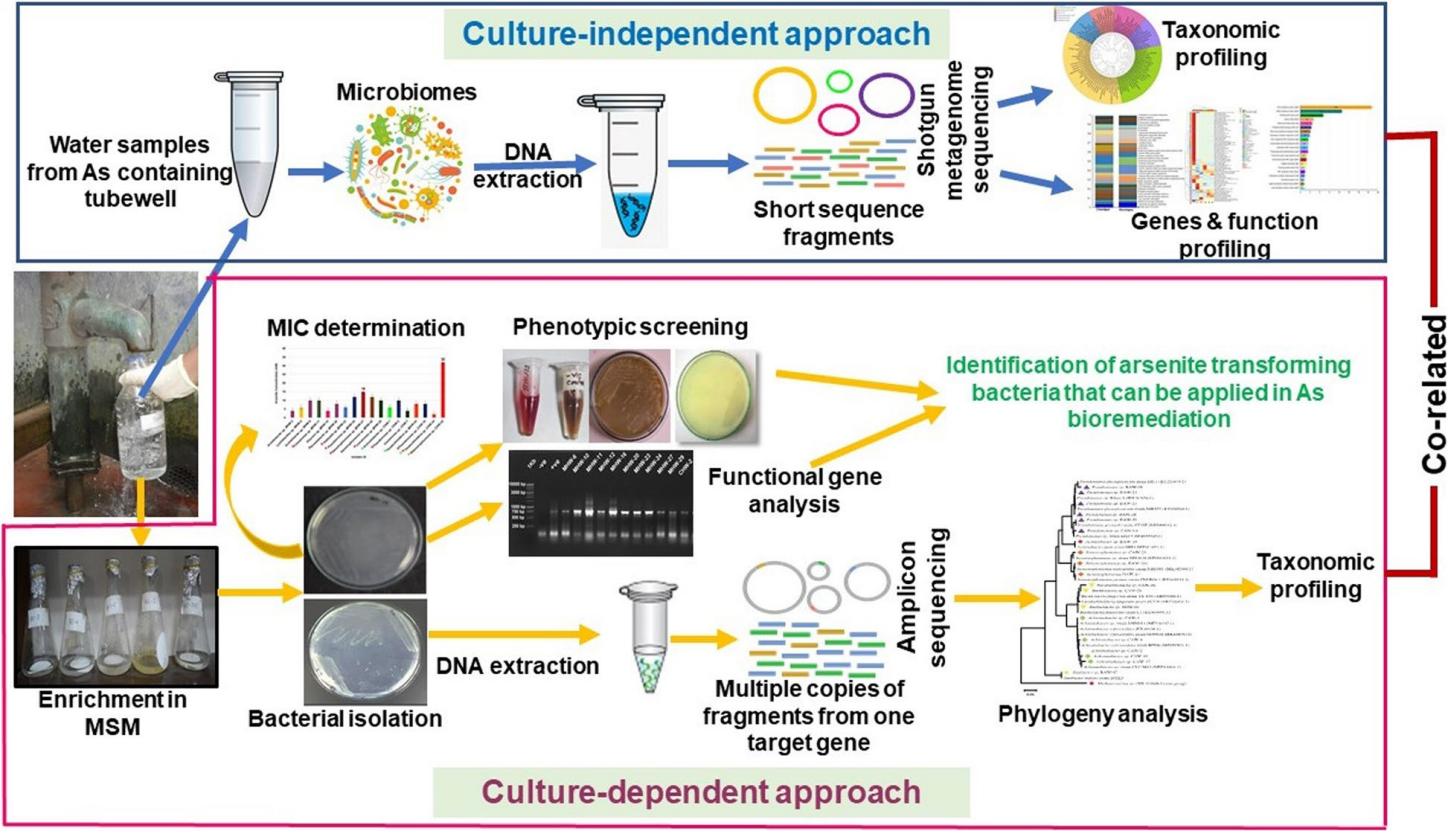
Graphical abstract showing overview of the study. This study was carried out to assess the diversity and transformation potentials of the arsenic-affected groundwater microbiomes using both culture-dependent and independent (shotgun metagenomics) approaches

The last decade has seen an exponential growth in the availability of sequenced data [24–26], and with it the discovery and expansion of the known As transforming genes in microorganisms. Rapid advances in high-throughput next generation metagenomic sequencing and bioinformatics pipelines have replaced culture-based methods for characterizing microbiota in various contexts in the past decade [27, 28]. This study combines both culture-independent high-throughput shotgun whole metagenome sequencing (WMS) and culture-dependent approaches to explore active microbial community and novel functional genes involved in arsenic mobilization and detoxification in As-contaminated GW of two most arsenic-prone districts of Bangladesh (Fig. 1). This investigation will provide a complete insight into the phylogenetic diversity of groundwater arsenotrophic microbiome along with elucidation of their genetic determinants as well as scientific basis for mitigating arsenic pollution in the aquifers, soil and diverse epidemiological niches environment.

## Methods

### Study location, sample collection, and processing

We have selected two arsenic-prone districts viz. Munshiganj (23.5422° N, 90.5305° E) and Chandpur (23.2513° N, 90.8518° E) of Bangladesh (Fig. S1). Six (*n* = 6) groundwater (GW) samples have been collected from local tube wells using acid-washed and autoclaved collection bottles. Among them, four GW samples (M1 – M4) were collected from Munshiganj district and two samples (C1 – C2) from Chandpur district. After removing any flow-through water, approximately 3 L of GW from each tube well was collected and transported to the laboratory on ice (4ºC). One liter (1L) GW sample was acidified with 0.5 M HCl for hydrogeochemical analysis, and 2L was left untreated for anion and microbiological analysis. A Millipore membrane filtration unit (Millipore, Billerica, MA, USA) was used to vacuum filter approximately 250 mL of GW using 0.22 m nitrocellulose membrane filters with 45–97 mm. Following filtering, filters were kept at –20°C until DNA extraction [2].

### Hydrogeochemistry of As-contaminated groundwater

Immediately after collection of GW sample, temperature, pH, conductivity, DO (dissolved oxygen), TDS (total dissolved solids), total alkalinity, nitrite, and sulfate concentrations of the GW samples were measured using standard methods for water and wastewater examination using a potable waterproof Hanna multiparameter analyzer (Hanna HI9823 Multiparameter, USA) [2]. Anion (NO_2_^−^ and SO_4_^−^) concentrations were analyzed in untreated samples by Ion Chromatography (Shimadzu, USA). The total As content in the GW was determined using a flame atomic absorption spectrophotometer accompanied by a hydride generation system (Shimadzu, AA 7000, USA).

### Cultivation-dependent study of the As-contaminated groundwater microbiota

#### Enrichment and isolation of arsenite metabolizing bacteria

Each GW sample was filtered through a sterile 0.22 µm cellulose-nitrate filter (Osmonics, USA), and bacteria from the filter were enriched in 60 mL of a modified version of a minimal salt medium containing two mM NaAsO_2_ (Merck, Germany) previously described for autotrophic and heterotrophic arsenite oxidizing bacteria [8]. The enrichment broth was incubated aerobically at a temperature of 25°C and a speed of 120 rpm on a rotary shaker. After two weeks, ten milliliter of each enrichment medium was transferred to a 250 mL Erlenmeyer flask containing fifty milliliters of the respective enrichment medium. Thereafter, 100 µL of enrichment culture was diluted serially and spread onto autotrophic and heterotrophic minimal salts enrichment agar [2% (w/v)] plates containing 2 mM arsenite and incubated for seven days at 25°C [8]. Several colonies were isolated, purified, selected and stored at -80°C for further study.

#### Phenotypic screening of As (III) oxidation

We screened the isolates retrieved from As-affected GW for their capacity to convert arsenite (III) to arsenate (V). Arsenite transformation efficiency was determined using colorimetric methods (KMnO_4_ and AgNO_3_ assay) following previously developed protocols [8].

#### Detection of genetic determinants in arsenite [As (III)] tolerant isolates

Bacterial DNA from cultured arsenotrophs was extracted from using the Qiagen DNA Mini-Prep kit (Qiagen, USA) according to the manufacturer’s instructions. We used specific primers to conduct PCR for arsenotrophic functional genes [arsenite oxidizing gene (*aio*A) and arsenic resistance gene (*ars*B)] (Table 1).

**Table 1:** Primer sequences used for the detection of bacterial 16S rRNA gene, arsenite resistance gene, oxidizing gene, and corresponding annealing temperature used for PCR

Target gene	Primers	Sequences (5ʹ → 3ʹ)	Amplicon size (bp)	Annealing T (^o^C)	Reference
**16S** **rRNA**	27F	5′- AGAGTTTGATCCTGGCTCAG-3′	~ 1400- 1450	55	Hoque et al., 2020 [24, 27]
	1492R	5′- GGTTACCTTGTTACGACTT-3′			
***aio*A**	aoxBM1-2F	5’CACTTCTGCATCGTGGGNTGYGGNTA-3′	1100	52	Quemeneur et al., 2008 [15]
	aoxBM3-2R	5′- TGTCGTTGCCCCAGATGADNCCYTTYTC-3′			
***ars*B**	darsB1F	5′- GGTGTGGAACATCGTCTGGAAYGCNAC-3′	750	55	Achour et al., 2007 [29]
	darsB1R	5ʹ-CAGGCCGTACACCACCAGRTACATNCC-3ʹ			

#### Arsenite tolerance assay

The minimum inhibitory concentration (MIC) of As (III)) was calculated for 40 isolates (*n* = 40) including those that exhibited the presence of arsenotrophic (*aio*A and *ars*B) genes in functional gene PCR [2]. Each test was conducted twice to measure the MIC of the isolates. The isolates were grown in 5 mL of either heterotrophic or autotrophic broth medium at 30ºC and 120 rpm until the optical density at 600 nm reached 0.1. Each well of a 96-well microtiter plate was filled with 70 µL of concentrated heterotrophic broth medium supplemented with various doses of As (III) as NaAsO_2_ (0 to 32 mM) from a stock solution of 66.32 mM. Each well received 5 µL of bacterial inoculum (OD_600_ = 0.1). The remainder of the capacity is filled with autoclaved deionized water, resulting in a final volume of 100 µL for each well. Sodium arsenite solution and autoclaved deionized water were added to a concentrated heterotrophic broth medium to dilute it to the concentration required for routine use. One row was set up as a negative control with simply As (III) media (no inoculum). The microtiter plate was incubated at 30°C. After 24 h, we measured the initial cell density and bacterial growth using a spectrophotometer set to 600 nm.

#### Ribosomal (16S rRNA) gene sequencing and phylogeny construction

Seventeen isolates were selected randomly for bacterial ribosomal (16S rRNA) gene sequencing. Two universal primers, 27F (5′-AGAGTTTGATCCTGGCTCAG-3′) and 1492R (5′-GGTTACCTTGTTACGACTT-3′) were used to amplify the target gene fragments of the 16S rRNA (Table 1). Agarose gel electrophoresis (1.2% wt/vol) was used to verify the presence of PCR products (Fig. S2). DNA sequencing was carried out at First Base Laboratories Sdn Bhd (Malaysia) using Applied Biosystems highest capacity-based genetic analyzer (ABI PRISMR 377 DNA Sequencer) platforms with the BigDyeR Terminator v3.1 cycle sequencing kit chemistry [30]. Using Molecular Evolutionary Genetics Analysis (MEGA) version 7.0 for the larger datasets [31], the 16S rRNA gene sequences, amplified from all individual bacterial isolates, were aligned with each other and with relevant reference sequences obtained from the NCBI Database. A maximum-likelihood tree was generated by MEGA 7.0 software using default parameters, and visualized by iTOL v5.6.1 [32]. Nodal confidence in the resulting phylogenetic relationships was assessed using the bootstrap test (1000 replicates).

### Cultivation-independent (metagenomic) investigation of arsenic-contaminated groundwater microbiome

#### Genomic DNA extraction, metagenomic sequencing, and data processing

Total genomic DNA was extracted from As-contaminated GW samples following previously established protocol [8]. DNA quantity and purity were determined using NanoDrop ND-2000 spectrophotometer (ThermoFisher, USA) by measuring 260/280 absorbance ratio. Libraries (1 ng DNA/sample) for shotgun WMS were prepared with Nextera XT DNA Library Preparation Kit [33] according to the manufacturer’s instructions, and paired-end (2 × 150 bp) sequencing was performed using a NovaSeq 6000 sequencer (Illumina Inc., USA). Our metagenomic DNA yielded 228.73 million raw reads with an average of 38.12 million reads per sample (Data S1). The read quality of the resulting FASTQ files was reviewed and filtered using BBDuk (with parameters k = 21, mink = 6, ktrim = r, ftm = 5, qtrim = rl, trimq = 20, minlen = 30, overwrite = true) [34], and Illumina adapters, known Illumina artifacts, and phiX were removed [35]. Any sequences that fell below these cutoffs or readings that included multiple ‘N’s were discarded. After filtering the poor-quality reads, we found that 133.82 million reads (an average of 22.30 million reads per sample), and the overall GC content was 57%.

#### Microbiome characterization and concurrent functional analysis

The WMS data were analyzed using both open-source cloud-based metagenomic mapping based and assembly-based hybrid methods of IDSeq [36] and MG-RAST 4.0 (MR) [37], respectively. IDseq—an open-source cloud-based pipeline—was used to classify sequences having NTL (nucleotide alignment length in base pairs) more than 50 and an NT % identity greater than 97 [33]. In IDSeq analysis, a ‘target’ genome library was constructed containing all prokaryotic sequences from the NCBI Database. The WMS reads were then aligned against the target libraries using the very sensitive Bowtie 2 algorithm [38]. The raw sequences were simultaneously uploaded to the MR server with properly embedded metadata for metabolic functional assignment. We used minimum identity of ≥ 90% for metabolic functional analysis through KEGG (Kyoto Encyclopedia of Genes and Genomes) pathways in the MR pipeline.

#### Detection of the virulent and antimicrobial resistance genes

We utilized the virulence factor database (VFDB) 2019 [39] to identify VFGs (virulence factor associated genes) in the As-contaminated GW microbiomes. Each protein in each sample category was utilized as a query to search for similarities to VFG protein-coding features. We sought to identify the best hit (best-scored alignment) that permitted us to assign a VFG function to each metagenomic protein. We simultaneously used the ResFinder 2.0 database [40] to detect AMRGs in the microbiomes. To find the corresponding genes and/or protein families, the ResFinder database was incorporated into the AMR++ algorithm. The VFGs and AMRGs that met the following similarity criteria (cut off): e-value < 1e^−5^, percent identity ≥ 80%, alignment length/subject length ≥ 0.8, and alignment length/ query length ≥ 0.8 were included in the study. Thus, the number of distinct classes (gene families) found in a metagenome reflects the variety of VFGs and AMRGs characteristics [24, 27].

#### Statistical analysis

We used a pair-wise non-parametric Kruskal Wallis rank-sum test, with Bonferroni correction, to compare the relative abundances of identified microbial taxa in As-contaminated GW samples. Comparative metabolic functional profiling was done using prokaryotic reference metagenomes from the MR database [33]. To identify differentially abundant KEGG functions (at various KEGG orthologues; KOs), non-parametric Kruskal Wallis rank-sum tests were applied with IBM SPSS (SPSS, Version 23.0, IBM Corp., NY USA).

## Results

### Hydrogeochemical properties of the arsenic-contaminated groundwater

The hydrological and geochemical properties of the analyzed GW samples are summarized in Table 2. Temperature, pH, and dissolved oxygen (DO) levels were within acceptable limits for drinking water in all samples. The average temperature and pH of the As-contaminated GW samples were 27.33°C (range: 26.1 to 28.1°C; higher in Munshiganj), and 6.7 (range: 5.77 to 7.6; higher in Chandpur), respectively. The level of dissolved oxygen (DO) and total dissolved solids (TDS) in the As-contaminated GW samples also varied between two sampling areas, with an average of 3.17 and 444.84, respectively. All of the GW samples had an average As concentration of 0.23 mg/L (range 0.05 to 0.27 mg/L) exceeding the WHO and Bangladesh acceptable level of 0.01 mg/L and 0.05 mg/L, respectively, except one GW sample from the Munshiganj (M4; 0.05 mg/L) district (Table 2). By comparing the As concentrations in the GW samples between study regions, we found that GW samples from Chandpur district had higher mean As concentration (0.24 mg/L) than those of Munshiganj (0.178 mg/L) district. Furthermore, average concentrations of Fe (3.75 mg/L), Mn (0.67 mg/L), Ca (38.49 mg/L), and K (154.38 mg/L) were found to exceed the WHO permissible limit. Heavy metals (i.e., cadmium, mercury, selenium, vanadium, and antimony) were absent in all of the GW samples tested (Table 2).

**Table 2:** Hydrogeochemical characteristics of groundwater samples collected from Munshiganj and Chandpur district, Bangladesh

**Division**	**District** **and** **Upazila**	**Location** **(Union)**	**Coordinates**	**GW Sample**	**Depth of well (m)**	**T (**^**o**^**C)**	**pH**	**Conductivity** **(µS/cm)**	**DO**	**TDS**	**Alkalinity as CaCO**_**3**_** (mg/L)**	**Dissolved trace elements (mg/L)**						
												**As**	**Fe**	**Mn**	**Ca**	**K**	**NO**_**2**_^**−**^	**SO**_**4**_^**−**^
**Dhaka**	**Munshiganj** **(Srinagar Upazila)**	**Sholaghar**	23.5543° N, 90.2917° E	M1	103.63	28.1	6.45	473	3.43	220	250	0.27	4.97	0.267	40.47	70.12	< 3	< 4
				M2	121.92	28	6.5	419.67	4.36	264	241	0.19	7.24	0.184	30.80	91.64	< 3	< 4
		**Birtara**	23.5667° N, 90.3167° E	M3	97.536	27.9	6.42	567.5	2.9	298	250	0.20	8.39	0.291	5.46	151.29	< 3	< 4
				M4	243.84	27.8	5.77	1813.38	4.18	210	183.34	0.05	1.84	1.955	131.02	594.5	< 3	< 4
**Chattogram**	**Chandpur** **(Kachua Upazila)**	**Ashrafpur**	23.2781° N, 90.9773° E	C1	101.25	26.1	7.6	2.22	1.38	1228	ND	0.25	0.04	ND	21.14	9.213	4.26	< 4
				C2	95.63	26.1	7.54	850	2.8	449	ND	0.23	0.03	ND	2.03	9.52	3.70	< 4

ND: Not determined; WHO standard limit for drinking water (4^th^ Edition): Temperature: 30^0^C; pH: 7- < 8; Conductivity: ≤ 4 00 µS/cm Dissolve oxygen (DO): 5 mg/L; TDS: 500 mg/L to ≤ 1000 mg/L; Alkalinity: 200 mg/L to ≤ 600 mg/L; As: 0.05 mg/L for BD; Mn: .01 mg/L; Ca: 75 mg/L; K: 12 mg/L; Nitrite (NO_2_^−^): 3 mg/L; Sulfate (SO_4_^−^): 250 mg/L

### Screening and isolation of arsenotrophic bacteriome

We isolated 72 bacteria from heterotrophic (36 isolates) and autotrophic (36 isolates) enrichment culture media based on their colony characteristics. We screened As (III) oxidation ability of these isolates phenotypically. The amount of As transformed by the cultured isolates in both heterotrophic and autotrophic media was determined using a permanganate (KMnO_4_) colorimetric assay. The KMnO_4_ formed a brown precipitate in qualitative reaction with AgNO_3_. In this study, only 11 isolates demonstrated a positive result for As (III) transformation using the phenotypic assay. Isolates that were positive in the KMnO_4_ test (*n* = 11) were further investigated to verify their conversion efficiency of As through the silver nitrate test.

### Molecular markers involved in arsenic metabolism

Arsenotrophic gene profiling showed the existence of critical genetic factors governing the As geochemical cycle in the GW bacteriome. Therefore, both *ars*B and *aio*A genes coexisted in the bacteriome of As-contaminated GW. The molecular features and presence of *ars*B and *aio*A genes in the cultured bacteria (or isolates) is shown in Table 3. In this study, twenty-three isolates were found to harbor *ars*B gene. Of them, 11 isolates including *Comamonas* spp., *Klebsiella* spp., *Pseudomonas* spp., *Stenotrophomonas* spp. and *Pseudomonas* spp. were obtained from heterotrophic enrichment culture, and 12 isolates including *Pseudomonas* spp., *Paraburkhulderia* spp. and *Stenotrophomonas* spp. were obtained from autotrophic enrichment culture. Additionally, we detected ten *aio*A gene possessing bacteria such as *Paraburkhulderia* spp. and *Stenotrophomonas* spp. from autotrophic enrichment media. However, only *Achromobacter* spp., detected in heterotrophic culture medium, was found to harbor the *aio*A gene (Table 3 and Supplementary Table 1).

**Table 3:** Phenotypic and genotypic profiling of sequenced bacteria isolated from arsenic containing tubewell water of Munshiganj and Chandpur district

Type of enrichment culture	Sequenced isolates	Phylogenetic identification	Taxonomic class	As (III) transformation ability		Arsenotrophic genes		MIC (mM)
				**KMnO**_**4**_	**AgNO**_**3**_	**Oxidation** **(*aio*A)**	**Resistance** **(*ars*B)**	
**Heterotrophic**	MHW-2	*Acinetobacter sp*.	γ-proteobacteria	***-***	***-***	***-***	***-***	4
	MHW-4	*Lysinibacillus sp.*	Firmicutes	***-***	***-***	***-***	***-***	6
	MHW-8	*Acinetobacter sp*.	γ-proteobacteria	**-**	**-**	**-**	**-**	10
	MHW-11	*Comamonas sp*.	β- proteobacteria	***-***	***-***	**-**	** + **	10
	MHW-19	*Kluyvera sp.*	γ-proteobacteria	**-**	**-**	**-**	**-**	4
	MHW-23	*Klebsiella sp*.	γ-proteobacteria	**-**	**-**	**-**	** + **	6
	MHW-24	*Pseudomonas sp*.	γ-proteobacteria	**-**	**-**	**-**	** + **	12
	MHW-27	*Stenotrophomonas sp*.	γ-proteobacteria	**-**	**-**	**-**	** + **	15
	MHW-28	*Stenotrophomonas sp*.	γ-proteobacteria	**-**	**-**	**-**	**-**	12
	MHW-29	*Comamonas sp*.	β- proteobacteria	**-**	**-**	**-**	** + **	10
	CHW-1	*Achromobacter sp*.	β- proteobacteria	** + **	** + **	** + **	**-**	6
	CHW-2	*Pseudomonas sp.*	γ-proteobacteria	**-**	**-**	**-**	** + **	10
	CHW-7	*Comamonas sp*.	β- proteobacteria	**-**	**-**	**-**	**-**	4
**Autotrophic**	MAW-24	*Parburkholderia sp*.	β- proteobacteria	** + **	** + **	** + **	**-**	8
	CAW-10	*Pseudomonas sp.*	γ-proteobacteria	**-**	**-**	**-**	** + **	8
	CAW-24	*Parburkholderia sp*.	γ-proteobacteria	** + **	** + **	** + **	** + **	2
	CAW-25	*Stenotrophomonas sp*.	γ-proteobacteria	** + **	** + **	** + **	** + **	32

*MHW* Munshiganj heterotrophic water, *MAW* Munshiganj autotrophic water, *CHW* Chandpur heterotrophic water, *CAW* Chandpur autotrophic water

### Arsenite tolerant bacteria and their phylogenetic relationship

**Fig. 2:**
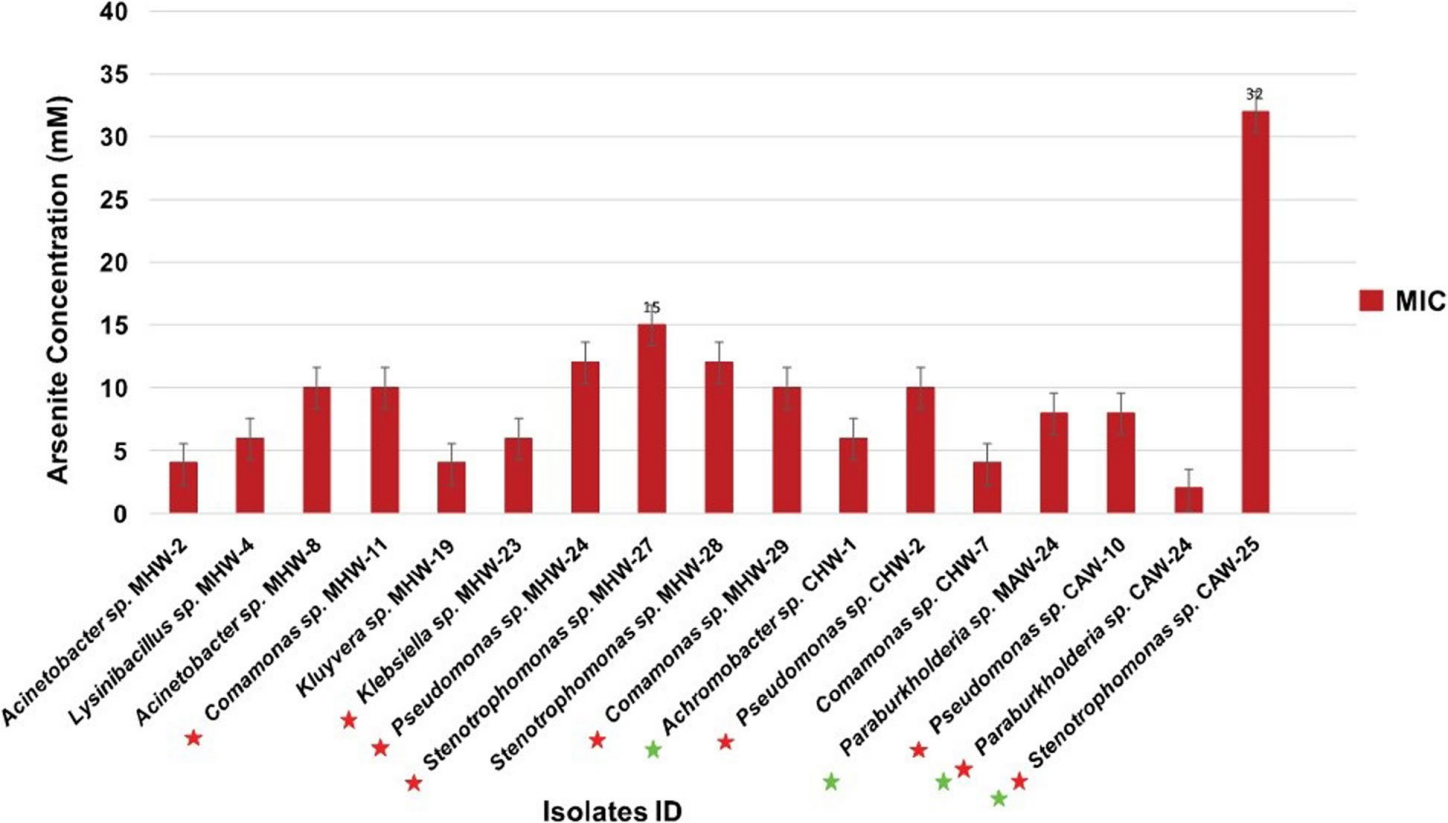
Minimum inhibitory concentration (MIC) of the arsenite tolerant bacteria. The MIC values detected ranged from 4 32 mM. Red and green stars indicate the presence of arsenite efflux pump (*ars*B) and arsenite oxidase (*aio*A) genes in the isolated bacteria, respectively. Each bar plot (deep maroon color) indicated the MIC (mM) value of each genus

**Fig. 3:**
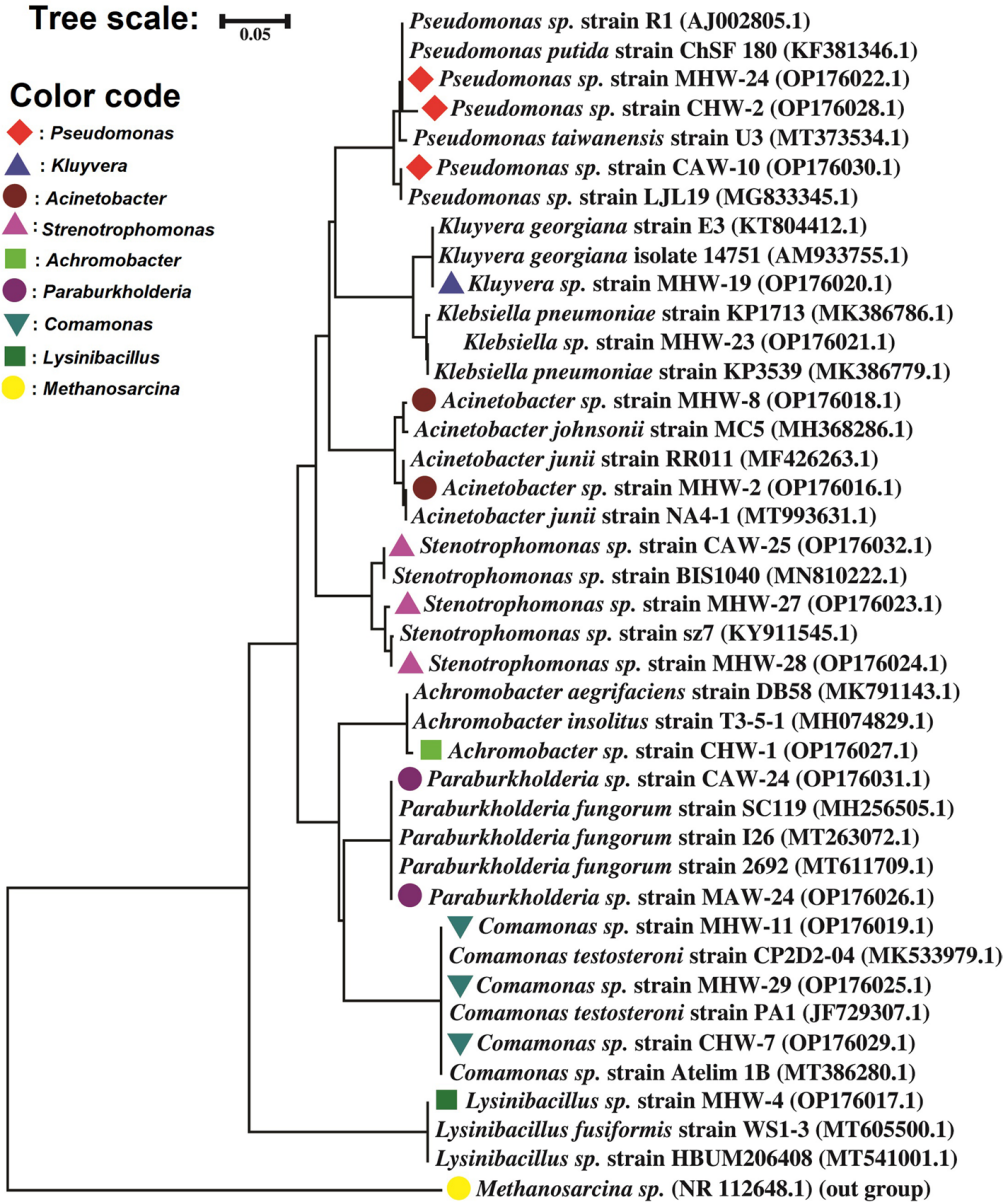
Phylogenetic tree of 16S rRNA gene sequences of arsenite tolerant groundwater bacteria. The maximum-likelihood tree was generated by MEGA 7.0 software, and visualized by iTOL v5.6.1. Nodal confidence in the resulting phylogenetic relationships was assessed using the bootstrap test (1000 replicates). *Methanosarcina* spp. was used as an outgroup. Different color codes (red: *Pseudomonas*, blueberry: *Kluyvera*, copper: *Acinetobacter*, violet: *Strenotrophomonas*, leafy green: *Achromobacter*, purple: *Paraburkholderia*, cyan: *Comamonas*, dark green: *Lysinibacillus*, and yellow: *Methanosarcina*) indicated different genera. Each reference and isolated strain’s GenBank accession number is displayed after the strain name

Forty isolates exhibited a broad range (2 mM to 32 mM) of tolerance to As (III). On an average, the autotrophic bacteria demonstrated higher tolerance to As (III) than the heterotrophic ones. The highest tolerance (32 mM) to As (III) was exhibited by *Stenotrophomonas* spp. strain CAW-25 while the lowest tolerance (2 mM) was found to *Parburkholderia* spp. strain CAW-24 (Fig. 2). The phylogenetic analysis based on the comparison of 16S rRNA gene sequences obtained in this study (*n* = 17) with closely related sequences deposited in GenBank of the NCBI database is shown in Fig. 3. The isolates of the As-contaminated GW samples belonged to nine polymorphic groups with phylogenetically diverse genera of *Pseudomonas* (3 isolates), *Stenotrophomonas* (3 isolates), *Comamonas* (3 isolates), *Acinetobacter* (2 isolates), *Paraburkholderia* (2 isolates), *Achromobacter* (1 isolate), *Klebsiella* (1 isolate), *Lysinibacillus* (1 isolate) and *Kluyvera* (1 isolate) (Fig. 3).

### Metagenomic investigation of arsenic-contaminated groundwater microbiome

#### Microbiome diversity and community structure

**Fig. 4:**
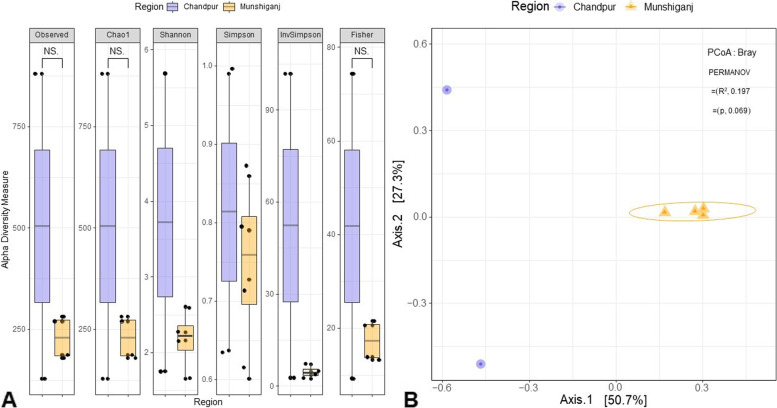
Diversity of microbiomes in arsenic-contaminated groundwater. **A** Box plots illustrating within-sample (alpha) microbial diversity. Alpha diversity measured on the Observed species, Chao1, ACE, Shannon, Simpson, and InvSimpson indices showed significant variations (*p* = 0.013, Kruskal–Wallis test). **B** Principal coordinate analysis (PCoA) (measured on the Bray–Curtis distance method) showing significant microbiome diversity in different samples i.e., Chandpur and Munshiganj (*p* = 0.001, Kruskal–Wallis test)

The WMS data yielded 133.82 million quality reads with an average of 22.30 million reads per sample (maximum = 22.87 million, minimum = 20.76 million). Through IDseq analysis, 61.03% of these WMS reads were found to correspond to prokaryotic (bacteria, archaea, and viruses) genomes in the reference sequence (RefSeq) database (https://www.ncbi.nlm.nih.gov/refseq/about/). The Observed species, Chao1, ACE, Shannon, Simpson, and InvSimpson diversity indices were used to calculate the microbial alpha diversity (i.e., within-sample diversity). The alpha diversity as measured on Observed species, Chao1, ACE, Shannon, Simpson, and InvSimpson diversity indices showed that GW samples of Chandpur district had significantly (*p* = 0.013, Kruskal Wallis test) higher within sample diversity than those of Munshiganj district (Fig. 4A). Additionally, we found significant changes in the microbial community structure between the study regions (*p* = 0.001, Kruskal Wallis test) (i.e., beta diversity analysis). At the species level, principal coordinate analysis (PCoA) revealed a clear segregation of samples between Chandpur and Munshiganj districts (Fig. 4B).

**Fig. 5:**
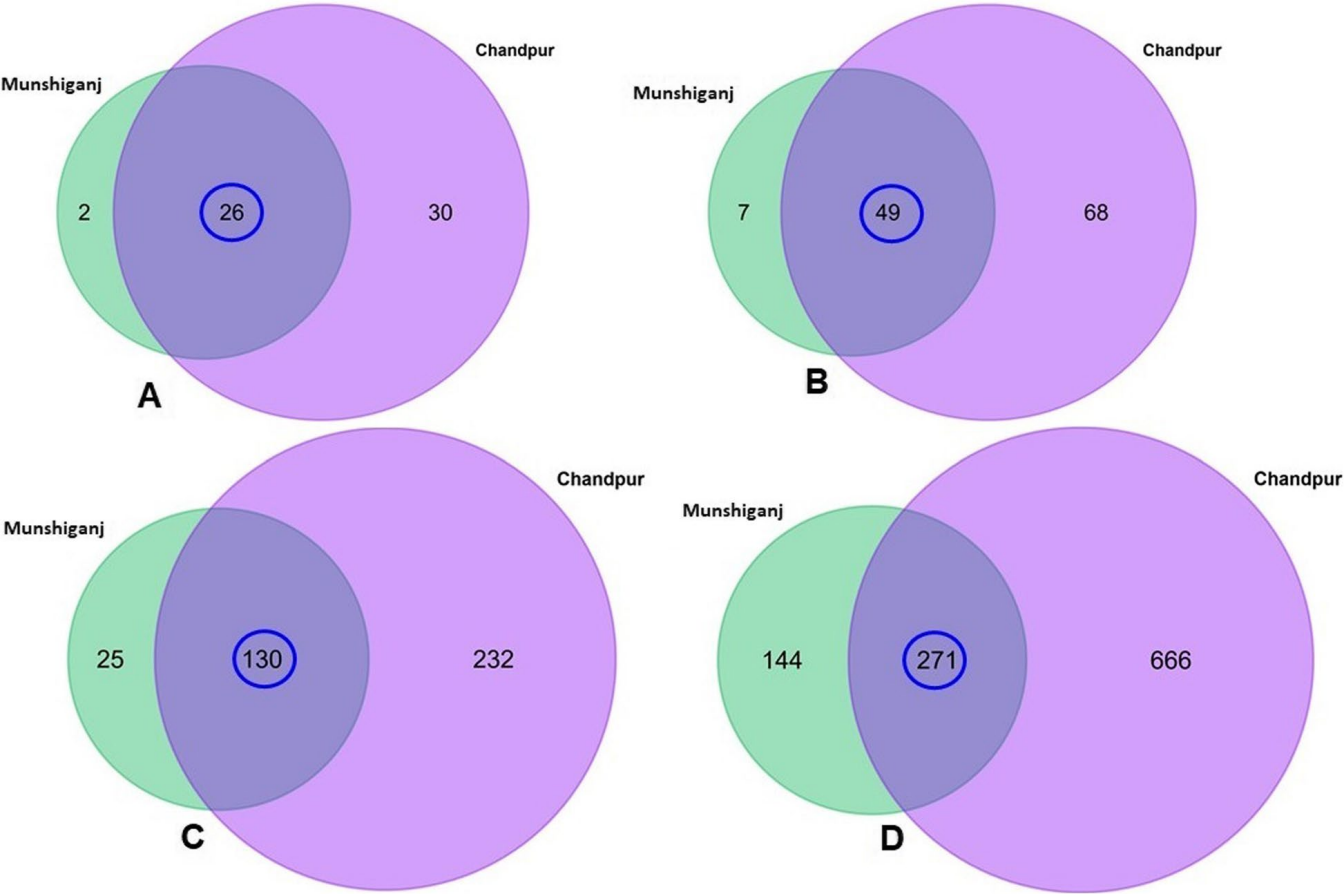
Taxonomic composition of microbiomes. Venn diagrams illustrate the unique and shared bacterial genomes in the arsenic-contaminated ground water samples of Munshiganj and Chandpur. **A** Venn diagram comparison of bacteria at an order level, **B** Venn diagram showing unique and shared bacterial families, **C** Shared and unique bacterial genera distribution between Munshiganj and Chandpur, and **D** Venn diagrams representing unique and shared species of bacteria two study areas. The blue circle indicates the microbiota that was shared between the study locations

Bacteria were the most abundant microbial domain in all samples, accounting for 98.56% of the total reads followed by *archaea* (0.83%) and viruses (0.26%). We identified 15, 60, 126, 387, and 1081 bacterial phyla, orders, families, genera, and species (Fig. 5), and the relative abundance of microbiomes varied considerably (*p* = 0.001, Kruskal Wallis test) across two study regions (Munshiganj versus Chandpur). Simultaneously, 59 archaeal and 55 viral genera were identified in this investigation. The composition and relative abundances of microbial taxa in both domains (archaea and virus) also varied considerably (*p* = 0.027, Kruskal Wallis test) across the two study sites.

#### Arsenic-contamination alters the bacteriome profile in groundwater

**Fig. 6:**
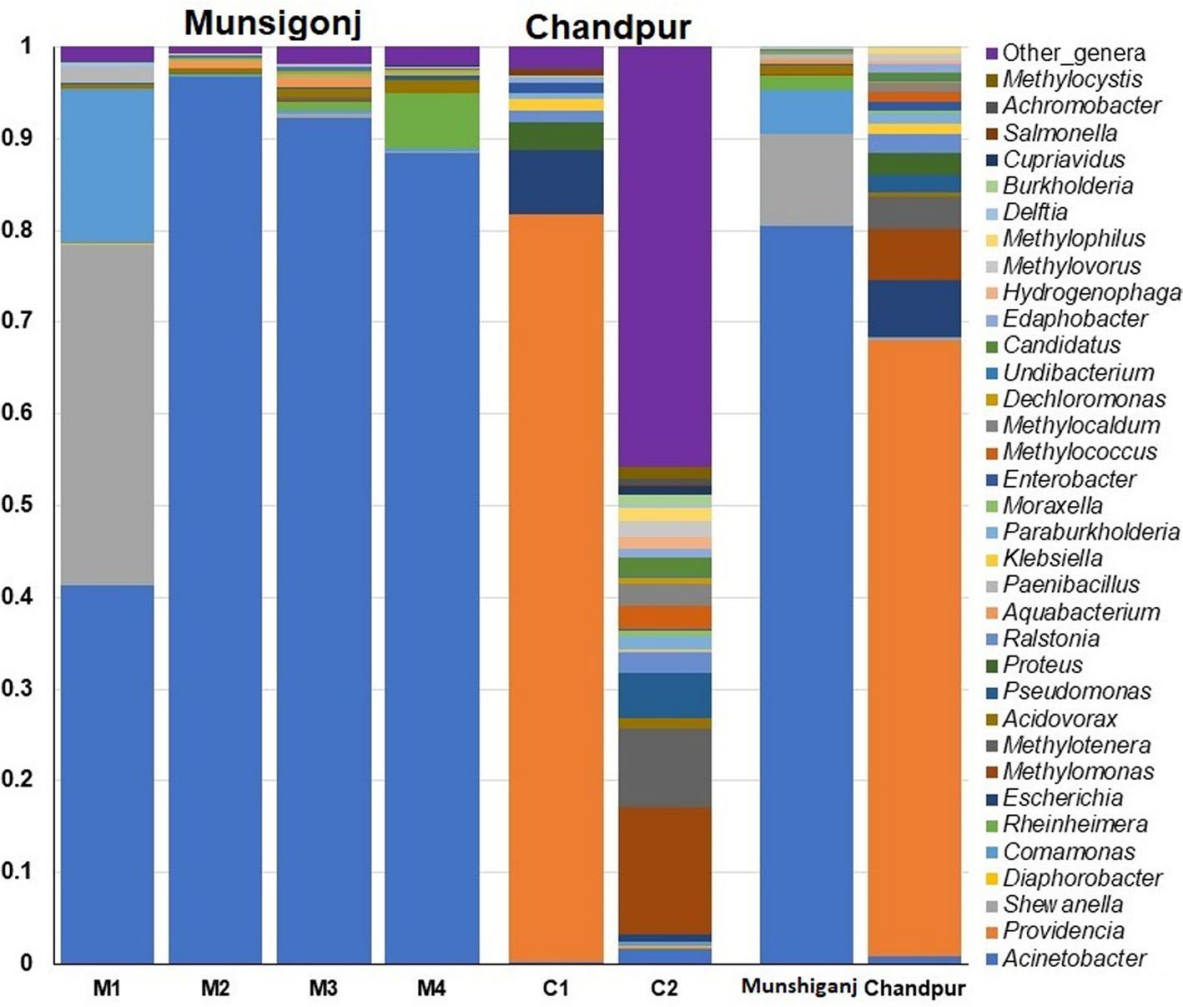
The taxonomic profile of the top 35 bacterial genera found in arsenic-contaminated groundwater. The 35 most prevalent bacterial genera are listed in order of decreasing relative abundance in six samples, with the remaining genera classified as ‘Other genera.’ Each stacked bar plot indicates the abundance of bacteria in the relevant category of samples. In contrast, the last two bar graphs represent the total relative abundance of bacterial taxa in Munshiganj and Chandpur district GW samples

To elucidate the changes in bacterial community and associated relative abundances in the As-contaminated GW of two study sites (e.g., Munshiganj and Chandpur), we identified the bacterial taxa up to species level. *Proteobacteria*, *Firmicutes*, and *Acidobacteria* dominated the As-contaminated GW samples of both metagenomes, accounting for > 99.5% of overall bacterial abundances. *Proteobacteria* was the most prevalent phylum, with a relative abundance of 98.84% in Munshiganj and 99.92% in Chandpur GW samples. We found 58 bacterial orders, of which 44.83% orders were found to be shared between the study locations (Fig. 5A). Out of 126 bacterial families detected, 56 and 117 families were detected in GW samples of Munshiganj and Chandpur districts, respectively, with 38.89% of bacterial families being shared between the two locations (Fig. 5B). By comparing the identified bacterial genera (*n* = 387) between two study sites, 155 and 362 genera were found in the GW samples of Munshiganj and Chandpur districts, respectively, with 33.60% shared genera (Fig. 5C). The current microbiome study demonstrated notable differences in species count in metagenome-assembled genomes (MAGs) of the As-contaminated GW samples of the Munshiganj and Chandpur districts. For instance, 1,081 bacterial species were identified including 414 and 937 species in the GW samples of Munshiganj and Chandpur district, respectively. Among these species, only 25.0% remained shared in both districts (Fig. 5D). *Acinetobacter* (79.22%), *Shewanella* (9.90%), *Comamonas* (4.62%), and *Rheinheimera* (1.65%) were the mostly predominating genera in the GW samples of Munshiganj (Fig. 6, Data S1) whereas *Providencia* (44.44%), *Citrobacter* (18.87%), *Escherichia* (4.04%), *Methylomonas* (3.69%), *Methylotenera* (2.30%), *Proteus* (1.58%), *Ralstonia* (1.33%), and *Pseudomonas* (1.32%) were the predominating bacterial genera in the As-contaminated GW of Chandpur district. The rest of the genera detected in both areas had relatively lower mean abundances (< 1.0%) (Fig. 6, Data S1).

**Fig. 7:**
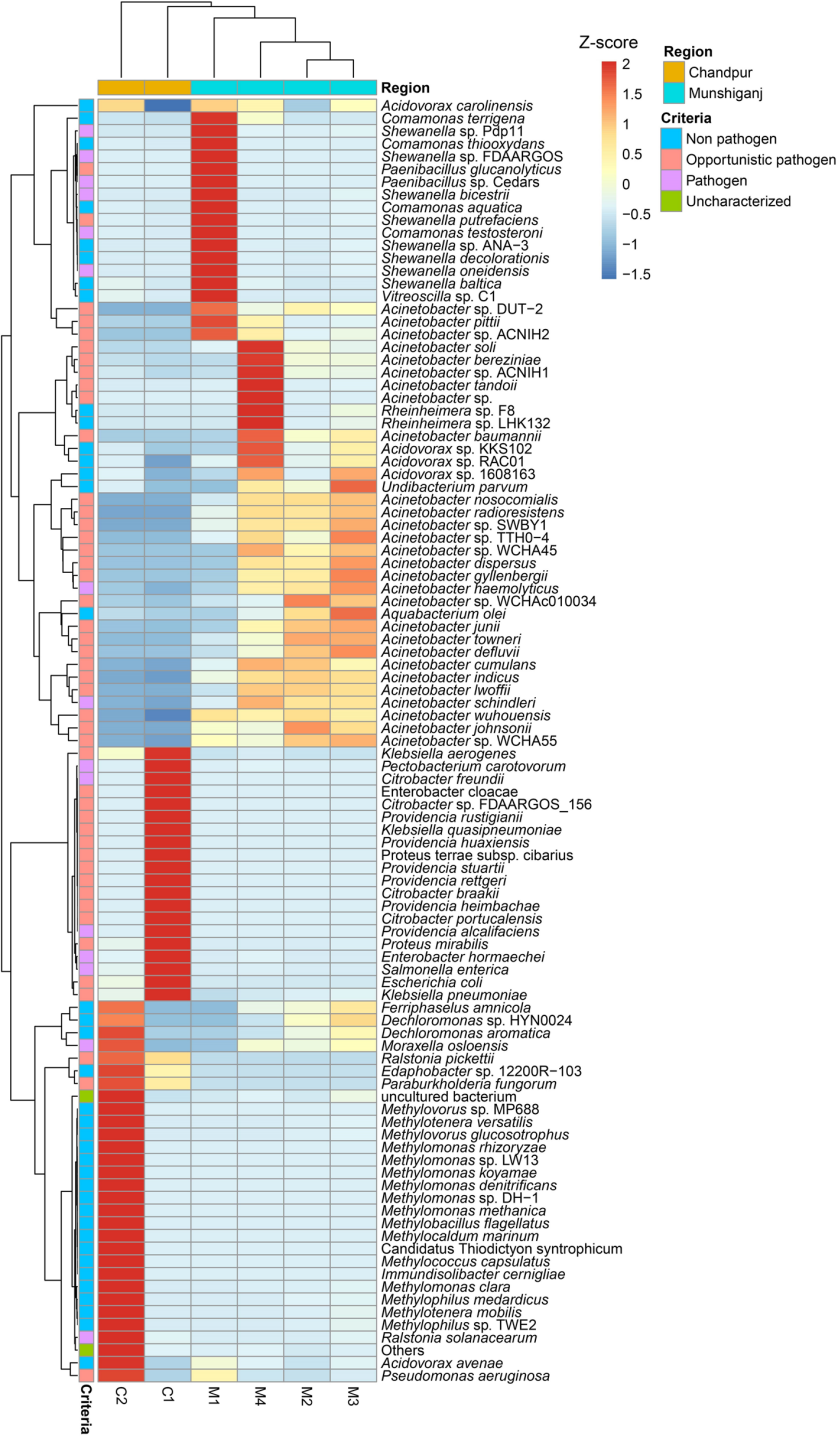
The species-level taxonomic profile of bacteria. The heatmap illustrates the hierarchical grouping of sample groups according to the relative abundance of the top 70 bacterial species revealed in the Munshiganj (M1-M4) and Chandpur GW metagenomes (C1-C2). The heatmap’s relative values (after normalization), shown by colors, represent the degree of bacterial species aggregation or content among samples based on the study region (Munshiganj and Chandpur), and criteria (pathogenic, opportunistic and non-pathogenic). The color bar (red to blue) depicts the row Z-scores (2 to -1.5), with red indicating high abundance and blue indicating low abundance. On the left, the color of the squares shows the relative number of bacterial species within each category. Additionally contains the distribution and relative abundance of the bacterial species found in the research metagenomes. The distribution and relative abundance of the bacterial species in the study metagenomes are also available in Data S1

We further investigated whether the relative abundances of bacteria at the species level varied between two sample locations. The GW samples from the Chandpur district possessed a significantly (*p* = 0.023, Kruskal Wallis test) higher number of bacterial species than those from Munshiganj. Of the detected species, 61.61% were found to be exclusively unique in the As-contaminated GW samples of Chandpur while only 13.32% were found to be solely associated with As-contaminated GW samples of Munshiganj (Fig. 5D). The As-contaminated GW metagenome of the Munshiganj district was dominated by different species of *Acinetobacter* genus, such as *Acinetobacter johnsonii* (41.30%) and *A. junii* (11.54%), *A. baumannii*(8.54%), *Acinetobacter* sp. WCHA45 (4.50%), *A. tandoii* (3.67%), *A. pittii* (1.71%), and *Acinetobacter* sp. WCHA55 (1.10%). Other predominant species in the GW samples of Munshiganj were *Shewanella* sp. 354 (8.53%), *Comamonas thiooxydans* (2.64%), *Rheinheimera* sp. F8 (1.50%), and *C. testosteroni* (1.21%) (Fig. 7). Conversely, *Providencia alcalifaciens* (41.17%), *Citrobacter portucalensis* (13.60%), *Escherichia coli* (3.97%), *Citrobacter freundii* (3.40%), *Methylotenera mobilis* (1.31%), *Proteus cibarius* (1.14%), and *Ralstonia pickettii* (1.06%) were the top abundant species in the GW samples of Chandpur district (Fig. 7). Rest of the bacterial species detected in both metagenomes had relatively lower abundances (< 1.0%), which varied remarkably between the study sites (Fig. 7, Data S1).

One of the hall mark findings of the present study was the detection of pathogenic, opportunistic, non-pathogenic, and uncharacterized bacterial species in both sample groups. According to the pathogenicity profile, 38.0% bacterial species were non-pathogenic irrespective of study locations, whereas 46.0%, 14.0, and 2.0% were opportunistic, pathogenic, and uncharacterized, respectively (Fig. 7). Among the pathogenic bacteria, *Providencia alcalifaciens* (11.34%), *Shewanella* sp. 354 (6.17%), *E. coli* (1.11%), *C. freundii* (0.94%), and *A*. *haemolyticus* (0.68%) were the top abundant species. Likewise, *A. johnsonii* (29.93%), *A. junii* (8.34%), *A. baumannii* (6.19%), *C. portucalensis* (3.75%), *Acinetobacter* sp. WCHA45 (3.25%), *A. tandoii* (2.65%), *A. pittii* (1.24%), *Acinetobacter* sp. WCHA55 (0.78%) and *Acinetobacter* sp. ACNIH2 (0.53%) predominately opportunistic pathogens (Fig. 7, Data S1).

#### Arsenic-contamination associated changes in archaeal and viral communities in the groundwater

**Fig. 8:**
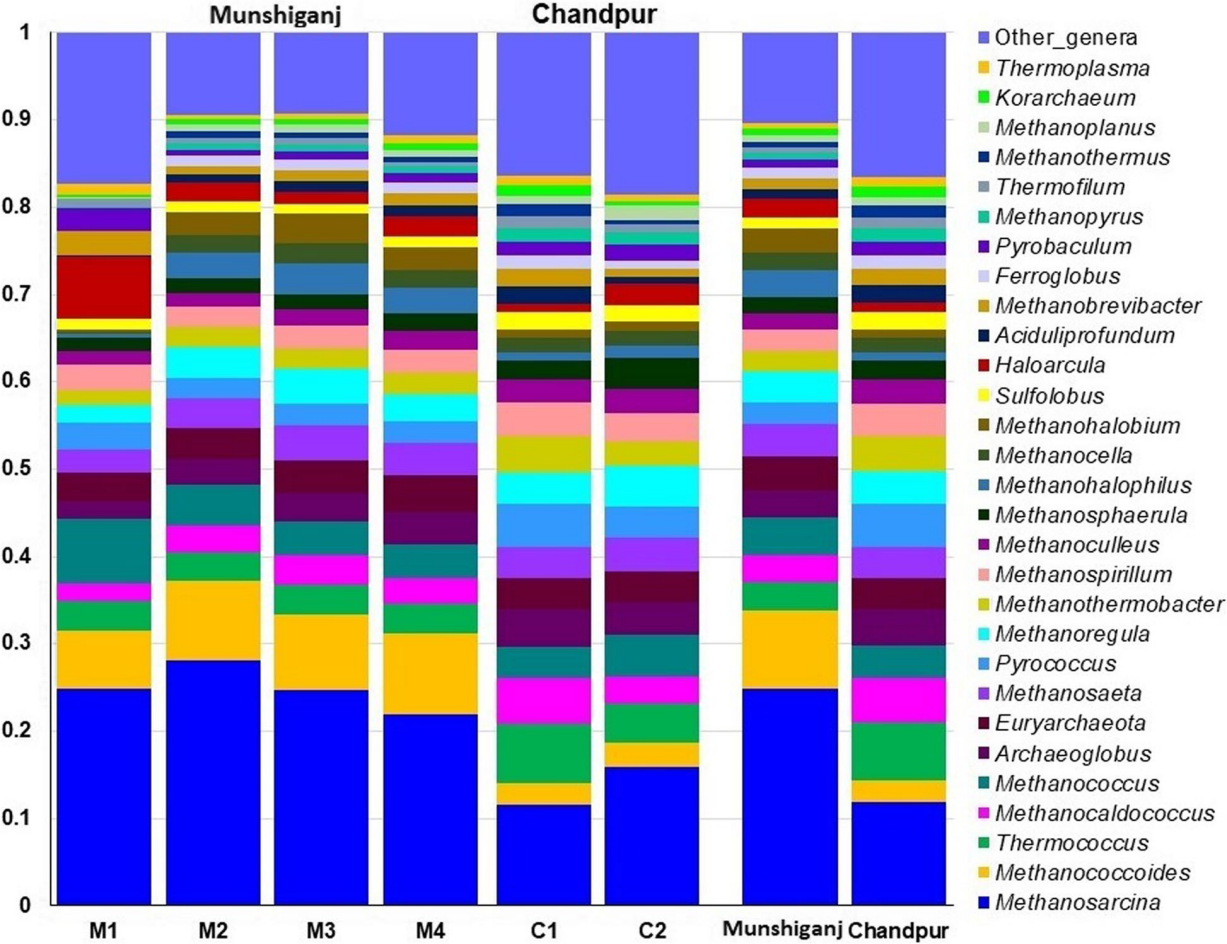
The taxonomic composition of the top 30 archaeal genera in arsenic-contaminated groundwater. The 29 most prevalent archaeal genera are listed in order of decreasing relative abundance in six samples, with the remaining genera classified as ‘Other genera.’ Each stacked bar plot indicates the abundance of archaea in the respective samples category. In contrast, the last two bar graphs represent the total relative abundance of archaeal genera in Munshiganj (M1- M4) and Chandpur (C1- C2) district GW samples

Another noteworthy finding of this study is the detection of archaeal (Fig. 8) and viral (Fig. S3) components of the microbiomes in As-contaminated GW samples of both study areas. The GW samples of Chandpur district were dominated by *Thermococcus* (6.96%), *Methanocaldococcus* (5.27%), *Pyrococcus* (5.04%), *Archaeoglobus* (4.35%), *Methanothermobacter* (4.24%), *Methanospirillum* (3.90%), *Methanoculleus* (2.80%), *Methanosphaerula* (2.30%), *Sulfolobus* (2.08%), and *Aciduliprofundum* (2.06%) archaeal (Fig. 8). Conversely, *Methanosarcina* (27.01%), *Methanococcoides* (9.10%), *Methanococcus* (4.56%), *Methanoregula* (3.90%), *Methanosaeta* (3.82%), *Methanohalophilus* (3.20%), *Methanohalobium* (3.03%), *Methanocella* (2.14%), and *Haloarcula* (2.13%) were the most abundant archaeal genera in As-contaminated GW samples of the Munshiganj district (Fig. 8). The rest of the archaeal genera had a relatively lower abundance (< 2.0%) in both study sites that varied significantly (*p* = 0.027, Kruskal Wallis test) between the sample categories.

The viral fraction of the current As-contaminated GW microbiomes was dominated mainly by the members of the *Siphoviridae* (44%), *Podoviridae* (28%), and *Myoviridae* (26%) families (Data S1). The predominating viral genera detected in As-contaminated GW samples of Chandpur district were *Siphovirus* (24.90%), *P2-like viruses* (11.33%), *Podovirus* (10.49%), *Lambda-like viruses* (9.40%), *N15-like viruses* (6.62%), *Myovirus* (7.82%), *Epsilon15-like viruses* (6.60%), and *P22-like viruses* (5.49%) (Fig. S3). The As-contaminated GW samples of Munshiganj, however, had a relatively higher abundance of *Siphovirus* (20.95%), *Myovirus* (13.71%), *P2-like viruses* (11.60%), *Bpp-1-like viruses* (10.27%), *Lambda-like viruses* (9.70%), and *Podovirus* (7.57%). The other viral genera identified in both metagenomes were relatively rare (5.0%) and differed considerably across sample locations (*p* = 0.027, Kruskal Wallis test) (Fig. S3, Data S1).

#### Virulence and antimicrobial resistance profile of the arsenic-contaminated groundwater microbiomes

We identified 92 VFGs comprising 69 and 61 in the As-contaminated GW samples of Chandpur and Munshiganj district, respectively. Though the composition of the VFGs varied between the study sites, their relative abundances did not differ significantly (*p* = 0.971, Kruskal Wallis test) between the sample categories. The most abundant VFG identified in microbiomes of both sites was *omp*A, which encodes for outer membrane proteins. By comparing the relative abundances of the rest of the VFGs among the microbiomes of both locations, we found that outer membrane protein; *omp*A (21.98%), biofilm regulation proteins; *bfm*R (12.83%), multidrug efflux pump; *acr*B (7.04%), sensor kinase; *bfm*S (4.03%), biofilm-associated protein; *bap* (3.61%), phosphoinositide signaling protein; *plc* (3.36%), efflux pump membrane transporter; *ade*G (3.09%), iron acquisition/ferric siderophore ABC transporter; *fep*A (2.44%), siderophore efflux system; *bar*B (2.29%) and enterobactin synthase component; *ent*B and *ent*E (2.12%) were the predominantly abundant virulence-related functional pathways/genes linked to arsenic contamination of the GW (Fig. S4). We also found that As-contaminated GW microbiomes were enriched with proteins involved in biofilm formation and control, signal transduction, multidrug efflux system, metabolism, siderophore transport, efflux system, and enterobactin synthase component (Fig. S4).

**Fig. 9:**
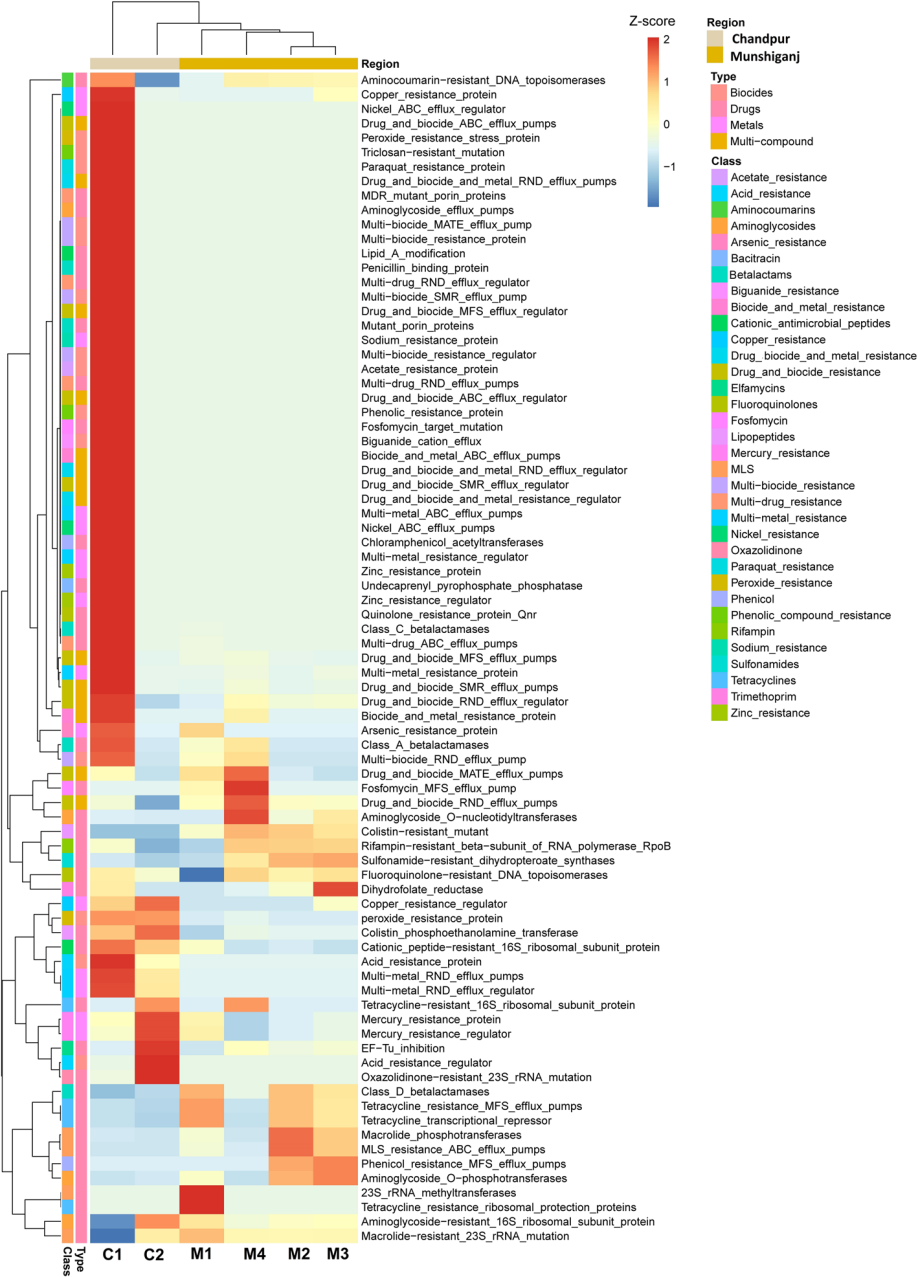
Antimicrobial resistance genes (AMRGs) detected in arsenic-contaminated GW microbiomes. Metagenome sequencing data was used to search for open reading frames (ORFs) compared against the ResFinder database to identify AMRGs with over 95% sequence identity. The relative values in the heatmap (after normalization), depicted by colors, indicate the aggregation degree or content of AMRGs in the samples according to study region (Munshiganj and Chandpur) and types (biocides, drugs, metals, and multi-compound). The color bar (red to blue) displays the row Z-scores (2 to -1): red color indicates high abundance; blue color represents low abundance. The color of the squares on the left shows the relative abundance of the respective AMRGs in each group

Simultaneously, we investigated the total number and classes of different antimicrobial resistance genes (AMRGs) present in the microbiomes. The categories and relative abundances of the AMRGs were significantly correlated (*p* = 0.0001, Kruskal Wallis test) with the relative abundance of the associated bacteria found in the samples of both regions (Fig. 9). We identified 81 AMRGs including 76 in the GM microbiomes of Chandpur and 41 in Munshiganj district. The detected AMRGs belonged to four types (biocides, drugs, metals, and multi-compounds) and 34 antibiotic classes. The macrolide-resistant 23S rRNA mutation (MLS23S) and aminoglycoside-resistant 16S ribosomal subunit proteins (A16S) were found as the predominantly abundant AMRGs, displaying higher relative abundances (46.23% and 24.54%, respectively) in As-contaminated GW microbiomes of Munshiganj than those of Chandpur (38.70% and 23.19%, respectively). These two AMRGs had several-fold higher relative abundances than the other AMRGs detected in both sample categories (Fig. 9, Data S2). Moreover, elfamycins (EF-Tu) inhibition (TUFAB; 5.83%), fluoroquinolone-resistant DNA topoisomerases (GYRA; 4.26%), cationic peptide-resistant 16S ribosomal subunit protein (CAP16S; 2.88%), arsenic resistance protein (ARSB; 1.14%), multi-metal resistance protein (ARSBM; 1.44%), drug biocide metal RND efflux pumps (CZCA; 1.53%), and fosfomycin target mutation (1.08%) were the other top abundant AMRGs in the GW microbiomes of Chandpur district (Fig. 9). Conversely, rifampin-resistant beta-subunit of RNA polymerase (RpoB; 7.91%) and drug biocide RND efflux_pumps regulator (CPXAR; 3.88%) were the top abundant AMRGs in the As-contaminated GW microbiomes of the Munshiganj district. The rest of the AMRGs also varied relative abundances between the two sample categories, more prevalent in the As-contaminated GW microbiomes of Chandpur district (Fig. 9, Data S2).

#### Distribution of arsenotrophic genes and other functional potentials of arsenic-contaminated groundwater microbiomes

Functional metabolic analysis of the same KEGG pathway/gene in As-contaminated GW microbiomes showed substantial changes in their relative abundances (*p* = 0.019, Kruskal Wallis test), connected with the divergence in microbiome diversity and composition (Fig. S5). Among the detected KO modules (*n* = 73), genes coding for arsenical pump-driving ATPase (24.90%), arsenic efflux pump protein (16.07%), methane metabolism (12.62%), arsenical-resistance protein ACR3 (12.55%), cytochrome c551 peroxidase (11.25%), arsenate reductase (9.61%), arsenite efflux pump ACR3, and related permeases (9.34%), superoxide dismutase [Fe] precursor (9.21%), arsenic resistance protein ArsH (7.44%), manganese superoxide dismutase (6.95%), Mg/Co/Ni transporter; MgtE (6.43%), arsenate reductase and related proteins, glutaredoxin family (5.88%), Na + /H + antiporter NhaD and related arsenite permeases (5.68%), arsenical resistance operon repressor (4.22%), ABC_transporter alkylphosphonate (5.27%) and arsenical resistance operon trans-acting repressor ArsD (3.22%) ABC transporter alkylphosphonate (5.27%) were top abundant among the microbiomes of GW metagenome of Chandpur district (Fig. S5). Conversely, arsenical-resistance protein ACR3 (21.14%), proteins for protection from ROS (19.60%), arsenate reductase (17.62%), ATP-dependent efflux pump transporter (14.09%), Co/Zn/Cd efflux system component (13.72%), citrate/TCA cycle (11.25%), oxidative stress (9.77%), oxidative phosphorylation (7.90%), arsenical resistance operon repressor (7.57%), and cell division (7.46%) associated genes were predominantly abundant in the microbiomes of Munshiganj metagenome (Fig. S5).

## Discussion

Microbial communities in natural environments play important roles in biogeochemical cycles. Arsenic contamination of GW and soil is a severe health risk in Southeast Asia, particularly in Bangladesh [2]. Developing an efficient and environment-friendly bioremediation technology requires knowledge on microbiome participating in As-metabolism within the As-contaminated GW [8]. In this study, the microbial consortia and related genes in As-contaminated GW were explored using culture-dependent and -independent (shotgun deep metagenomic) approaches. We also investigated the biotransformation potentials of the As-contaminated GW microbiome that will help researchers in designing a cost-effective and environmentally bioremediation model in future. Six samples were collected from As-contaminated GW samples of Munshiganj (*n* = 4) and Chandpur (*n* = 2) districts where the mean As concentrations exceeded the WHO recommended permissible limits of As for Bangladesh (> 0.05 mg/L). The mean As concentration was remained higher for GW samples of Chandpur district (0.24 mg/L) than those of Munshiganj (0.178 mg/L) district. The mean concentrations of Fe, Mn, Ca, and K were also found to exceed the WHO permissible limit. Remarkably, no heavy metal was detected in the As-contaminated GW samples. These findings corroborated with the finding of many previous studies conducted in Bangladesh [41, 42]. One of the most important focuses of this study was to isolate arsenotrophic bacteria (both arsenite tolerant and arsenite transforming) from As-contaminated GW samples and to explore their distribution and diversity. A total of 72 bacterial isolates were obtained from the six GW samples. Among these isolates, diverse taxonomic classes were detected. For instance, *Acienetobacteria, Achromobacter*, *Stenotrophomonas*, *Comamonas*, and *Pseudomonas* were the most dominating genera detected through heterotrophic enrichment culture (Fig. 3). The 16S rRNA partial gene amplification and sequencing revealed that As (III) tolerant isolates comprised of a mixture of various 16S rRNA genes indicating that it corresponds to an evolutionarily diverse bacterial consortium. In contrast, *Pseudomonas*, *Achromobacter*, and *Stenotrophomonas* were highly abundant among autotrophic bacteria. These bacterial genera are common and dominant inhabitants of GW worldwide [14, 43]. The bacterial genera detected in this study were also reported to reduce, oxidize, and methylate As [44, 45] and had been used in several bioremediation investigations for their broad metabolic capacities [2, 46]. Although, many of these genera have been reported earlier, but a few (e.g., *Kluyvera* spp. and *Lysinibacillus* spp.) are unique in this investigation.

We additionally investigated the functional gene diversity of the study isolates. It has already been reported that bacterial resistance to As results from energy-dependent efflux of either As (III) or As (V) from the cell through the As resistance (*ars*) operon [47]. The assay for existence of the pump-specific extrusion (*ars*B) and arsenite oxidizing (*aio*A) genes revealed that most of the isolates possessed these *genes* with an MIC range of 4 to 32 mM (Fig. 2). Previously, high levels of resistance were reported in As-resistant bacteria obtained from various As-contaminated habitats [11, 15]. GW In this study, the arsenite transporter *ars*B gene found in autotrophic *Stenotrophomonas* spp. showed the highest level of As (III) resistance (32 mM). The MIC of this bacterium was higher than the As-resistant *Stenotrophomonas maltophilia* S255 isolated previously from agricultural soil contaminated by industrial effluent [48]. Additionally, *Stenotrophomonas* spp*.* was reported as a novel arsenic hyper-resistant bacteria with an MIC of > 32 mM for arsenite isolated from Crven Dol mine [49]. However, in this investigation, autotrophic GW isolates were found to have more As (III) resistance than heterotrophic isolates. The energy-dependent efflux pump gene *ars*B strongly links to the MICs of arsenite [14, 49]. A series of earlier investigations demonstrated that Gram-negative bacterial cell walls are much more protective against toxic metals, developing high resistance in metal-contaminated environments compared to Gram-positive bacteria [50, 51]. Our observation is also compatible with this statement, and the most arsenite-tolerant strains in this study belonged to Gram-negative *Stenotrophomonas* spp., *Pseudomonas* spp. and *Acinetobacter* spp*.* etc. The presence of these bacteria suggests that they may play an important role in As-contaminated GW and/or soils due to their high adaptability to extreme environments, providing a stabilizing effect for the water functions [52, 53].

The shotgun WMS approach was used to understand the structural and functional diversity present in As-contaminated GW from two more As-prone districts (Chandpur and Munshganj) of Bangladesh. The contamination of drinking water from both As and microbial pathogens occurs in Bangladesh [2, 8]. A metagenomic investigation of the GW could thereby be able to provide information on the types of microbes present and help elucidate As metabolic pathways, and potential assay targets for monitoring the transfer of microbiomes (including opportunists and pathogens) through the surface-to-GW axis. Microbial alpha-diversity in the As-contaminated GW samples of the Chandpur district (mean As concentration: 0.24 mg/L) was higher than those in the GW samples of Munshiganj district (mean As concentration: 0.178 mg/L) (Fig. 4A). Beta diversity demonstrated the significant microbiological distinction between two locations (Chandpur and Munshiganj) (Fig. 4B). The higher diversity and abundances were observed at each taxonomic level examined. This was probably due to an evolutionary adaptation of some specific microbial taxa to the As contamination stress in the GW [54]. The microbiome diversity we found in this study corroborates with many of the earlier studies [54, 55] where higher diversity in arsenic-resistant bacteria was reported in higher arsenic-, chromium- and copper-contaminated environment (soil or water) than that in less contaminated environment. All of the GW samples were dominated by Proteobacteria (including Gamma-, Beta- and Alpha-proteobacteria). A number of previous studies in reported that Proteobacteria are frequently present in metal (e.g., chromium and As) contaminated sites and are capable of metal transformation [8, 10, 55]. The higher abundance of Proteobacteria is connected to their capacity to live in metal-contaminated and stressful conditions [8, 56].

The noteworthy findings of the present WMS study are the taxonomic profiling of bacteria at both the genus and species-level. We found significant variations in the structure and composition of microbiomes at the genus and species levels across the GW samples of both Munshiganj and Chandpur districts. *Acinetobacter* was the most prevalent genus followed by *Shewanella*, *Comamonas* and *Rheinheimera* in the As-contaminated GW of Munshiganj. In contrast, *Providencia*, *Citrobacter*, *Escherichia*, *Methylomonas*, *Methylotenera*, *Proteus*, *Ralstonia*, and *Pseudomonas* were the most dominating bacterial genera in the GW of Chandpur district (Fig. 6). The predominant bacterial genera detected in this study through shotgun WMS approach are consistent with previous results obtained from traditional and 16S rRNA sequencing methods [8, 11, 53].

One of the hallmark findings of the present WMS investigation is to decipher the possible association of the archaeal and viral fractions with bacterial communities in the As-contaminated GW. In comparison to bacteria, the relative abundance and diversity of archaea and viruses remain substantially lower (< 1.0%). The anaerobic methanogenic genus *Methanosarcina* dominates the archaeal portion of all of the GW samples of both metagenomes. The cross-kingdom multi-microbiome interaction was always dominated by bacteria. Previously, archaea and viruses were identified in the As-contaminated surface and well water with a lower quantity (0.4—1.2%) [10, 56] supporting our present findings. Although, the presence of archaea is ubiquitous and universal in natural environment, high As content can restrict their prevalence due to their sensitivity or lack of an ‘As’ detoxification systems [10].

We detected 92 VFGs and 81 AMRGs in the six GW samples. On an average, the As-contaminated GW microbiomes of Chandpur district harbored more VFGs and AMRGs compared to those of Munshiganj. The most common antibiotic classes in the microbiomes of both sample groups were macrolide and aminoglycoside resistance genes. Antibiotic resistance genes (macrolide, aminoglycoside, beta-lactamase etc.) were found in bacteria isolated from As-contaminated tube well water of Bangladesh [14]. Heavy metal contamination affects the co-selection and transmission of AMRGs in aquatic systems [57, 58]. Functional metabolic analysis through the KEGG pathway revealed the abundance and distribution of different proteins involved in As metabolism in the As-contaminated GW samples. In the As-contaminated GW samples of both districts, genes for arsenic metabolism, including arsenate reductase, along with those for arsenic resistance, were present. Moreover, we found that genes encoding enterobactin synthase components (*ent*B and *ent*E), ABC ferric transporter and siderophore efflux systems were also present in the GW microbiomes. These systems participate in As mitigation, iron chelation, and metal detoxification [59]. These genetic pathways may affect arsenic mobility and toxicity [56]. Interestingly, we found no genes associated with arsenite oxidation in the GW microbiomes. But we confirmed the presence of arsenite oxidizing bacteria and the gene responsible for As (III) oxidation in the cultured isolates. The possible reason behind this might be the low depth of microbiome sequencing or the very low abundance of microbiome harboring the genes related to arsenite oxidation. However, we employed the enrichment culture media to isolate desired arsenotrophic bacteria. Further investigation will be needed to unveil the actual reason.

## Conclusion

Arsenic pollution in groundwater and soil is a global threat especially in Bangladesh. Novel genes and enzymes involved in microbial arsenotrophy are reported from diverse habitats; Bangladesh is behind due to a lack of arsenic microbial ecology research expertise. This study elucidated the microbial community and features responsible for As metabolism in As-contaminated GW of Munshiganj and Chandpur districts of Bangladesh. Bacterial dominance over other domains was established by shotgun WMS approach in As polluted locations. This study revealed genes encoding As resistance proteins and siderophores that enable bacteria to acquire iron from arsenopyrite minerals, releasing arsenic into the environment. The high frequency of As resistance and oxidation genes discovered using a cultivation-dependent approach revealed native bacterial community is actively involved in mobilizing and detoxifying As in GW. Metagenomic and enrichment studies addressed arsenotrophic microbiomes and their functions in As biogeochemical transformation. Future research can be focused on genetic and proteomic analyses of indigenous isolates to build green *in-situ* bioremediation strategy for As-contaminated locations in Bangladesh.

### Supplementary Information

**Additional file 1: Fig. S1. **Arsenic contaminated environmental (groundwater and soil) sample collection sites (marked with circular colored pin). Three arsenic-prone districts in Bangladesh were selected for arsenotrophic bacteriome study: Munshiganj and Chandpur. They are denoted by orange and red pins. **Fig. S2. **Agarose gel electrophoresis (on 1% agarose gel) of PCR-specific amplicon of arsenite efflux pump gene (*ars*B) of arsenite tolerant groundwater isolates enriched from (a) heterotrophic and (b) autotrophic medium. Lane-1, 2, and 3 of (a) and (b) image indicates 1Kb (Promega, USA) DNA marker, negative control, and positive control. The other lanes of both images indicate isolate code**. Fig. S3.** The taxonomic structure of the most prevalent virus taxa in arsenic-contaminated groundwater samples. The heatmap depicts the distribution of viral genera in the Munshiganj (M1-M4) and Chandpur (C1-C2) district GW samples. The color coding reflects the presence and completeness of each viral gene, displayed as a value (Z score) ranging from -3 (low abundance) to 3 (high abundance). The green color represents the maximum abundance of the particular genes in each sample, while the purple color represents the lowest abundance.** Fig. S4. **Virulence factors associated genes (VFGs) detected in arsenic-contaminated GW microbiomes. The distribution of top abundant 20 VFGs found in the arsenic-polluted GW microbiomes. VFGs are represented by different colored bars according to their relative abundances. Error bars show significant differences in the relative abundances of the corresponding VFGs.** Fig. S5. **Analysis of the functional genomic potentials of arsenic-contaminated GW microbial community through KEGG pathways. Between two metagenomic groups, bar charts represent the distribution of the 40 genes related to the discovered metabolic functional potentials determined using KEGG pathway analysis (Chandpur and Munshiganj). Each stacked bar plot depicts the frequency of occurrence of KEGG pathways in the relevant category's samples (Chandpur and Munshiganj district).** Table S1.** Cultural and molecular features of potential arsenite resistant and oxidizing bacteria retrieved from Munshiganj and Chandpur district.

**Additional file 2: Data S1.** Taxonomic information of the microbiomes (structure and relative abundance). **Data S2.** Antibiotic resistance, virulence and metabolic functional genes related information of the microbiomes.

## Data Availability

The sequence data and related metadata reported in this paper are available in the repository of the NCBI database under Sequence Read Archive (SRA) submission under BioProject accession- PRJNA916093 (https://www.ncbi.nlm.nih.gov/bioproject/?term=PRJNA916093). Supplementary information supporting the findings of the study are available in this article as Figs. S1-S5, Data S1-S2, and Table S1.
