# Supplementary material for: Metagenomic and culture-dependent approaches unveil active microbial community and novel functional genes involved in arsenic mobilization and detoxification in groundwater
Source: BMC Microbiol. 2023 Aug 30;23:241. doi: 10.1186/s12866-023-02980-0 (PMC10466822; doi:10.1186/s12866-023-02980-0)
Supplement: Supplementary file 1 — Additional file 1: Fig. S1. Arsenic contaminated environmental (groundwater and soil) sample collection sites (marked with circular colored pin). Three arsenic-prone districts in Bangladesh were selected for arsenotrophic bacteriome study: Munshiganj and Chandpur. They are denoted by orange and red pins. Fig. S2. Agarose gel electrophoresis (on 1% agarose gel) of PCR-specific amplicon of arsenite efflux pump gene (arsB) of arsenite tolerant groundwater isolates enriched from (a) heterotrophic and (b) autotrophic medium. Lane-1, 2, and 3 of (a) and (b) image indicates 1Kb (Promega, USA) DNA marker, negative control, and positive control. The other lanes of both images indicate isolate code. Fig. S3. The taxonomic structure of the most prevalent virus taxa in arsenic-contaminated groundwater samples. The heatmap depicts the distribution of viral genera in the Munshiganj (M1-M4) and Chandpur (C1-C2) district GW samples. The color coding reflects the presence and completeness of each viral gene, displayed as a value (Z score) ranging from -3 (low abundance) to 3 (high abundance). The green color represents the maximum abundance of the particular genes in each sample, while the purple color represents the lowest abundance. Fig. S4. Virulence factors associated genes (VFGs) detected in arsenic-contaminated GW microbiomes. The distribution of top abundant 20 VFGs found in the arsenic-polluted GW microbiomes. VFGs are represented by different colored bars according to their relative abundances. Error bars show significant differences in the relative abundances of the corresponding VFGs. Fig. S5. Analysis of the functional genomic potentials of arsenic-contaminated GW microbial community through KEGG pathways. Between two metagenomic groups, bar charts represent the distribution of the 40 genes related to the discovered metabolic functional potentials determined using KEGG pathway analysis (Chandpur and Munshiganj). Each stacked bar plot depicts the frequency of occurrence o [file 12866_2023_2980_MOESM1_ESM.pdf]

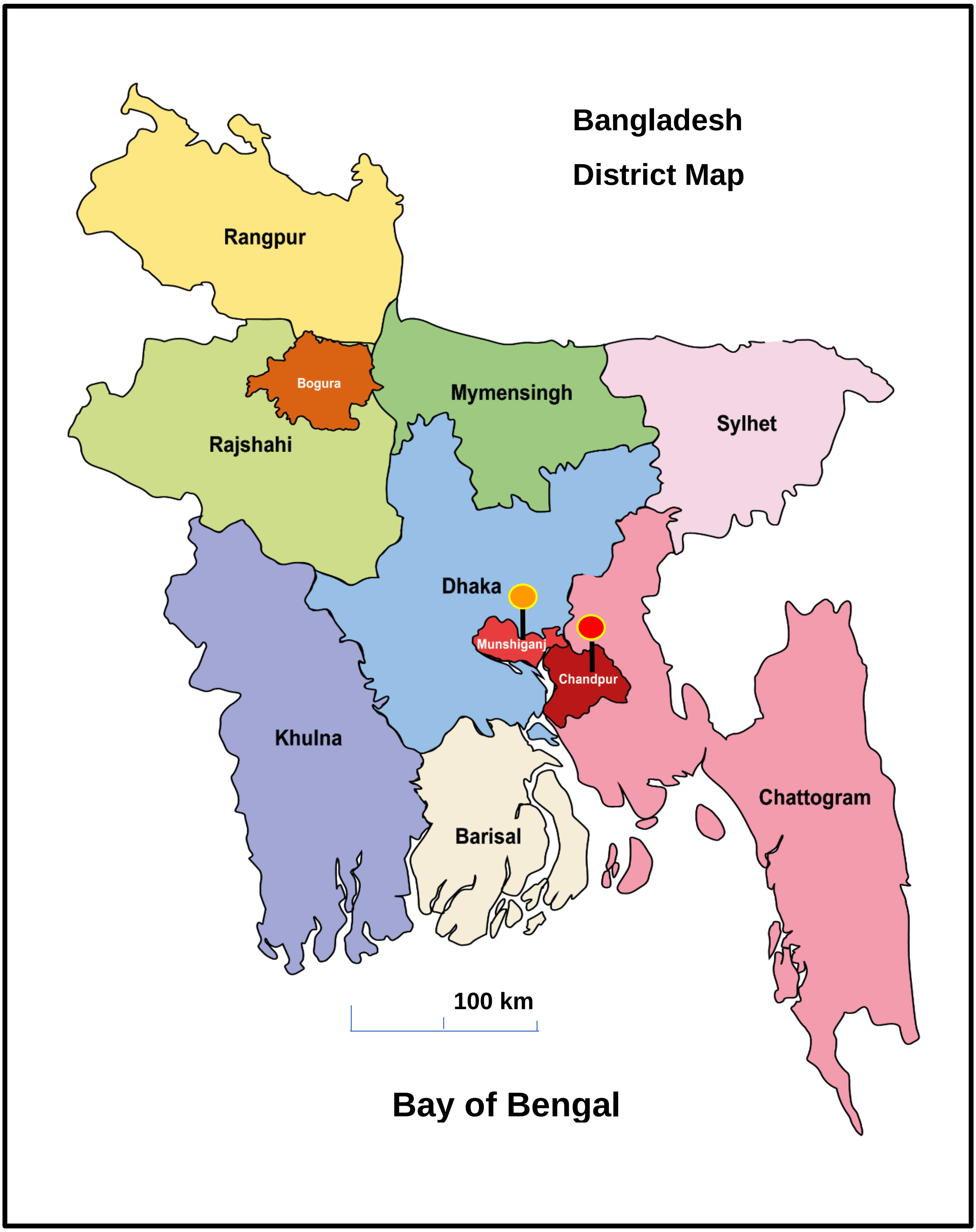

Fig. S1. Arsenic contaminated environmental (groundwater and soil) sample collection sites (marked with circular colored pin). Three arsenic-prone districts in Bangladesh were selected for arsenotrophic bacteriome study: Munshiganj and Chandpur. They are denoted by orange and red pins.

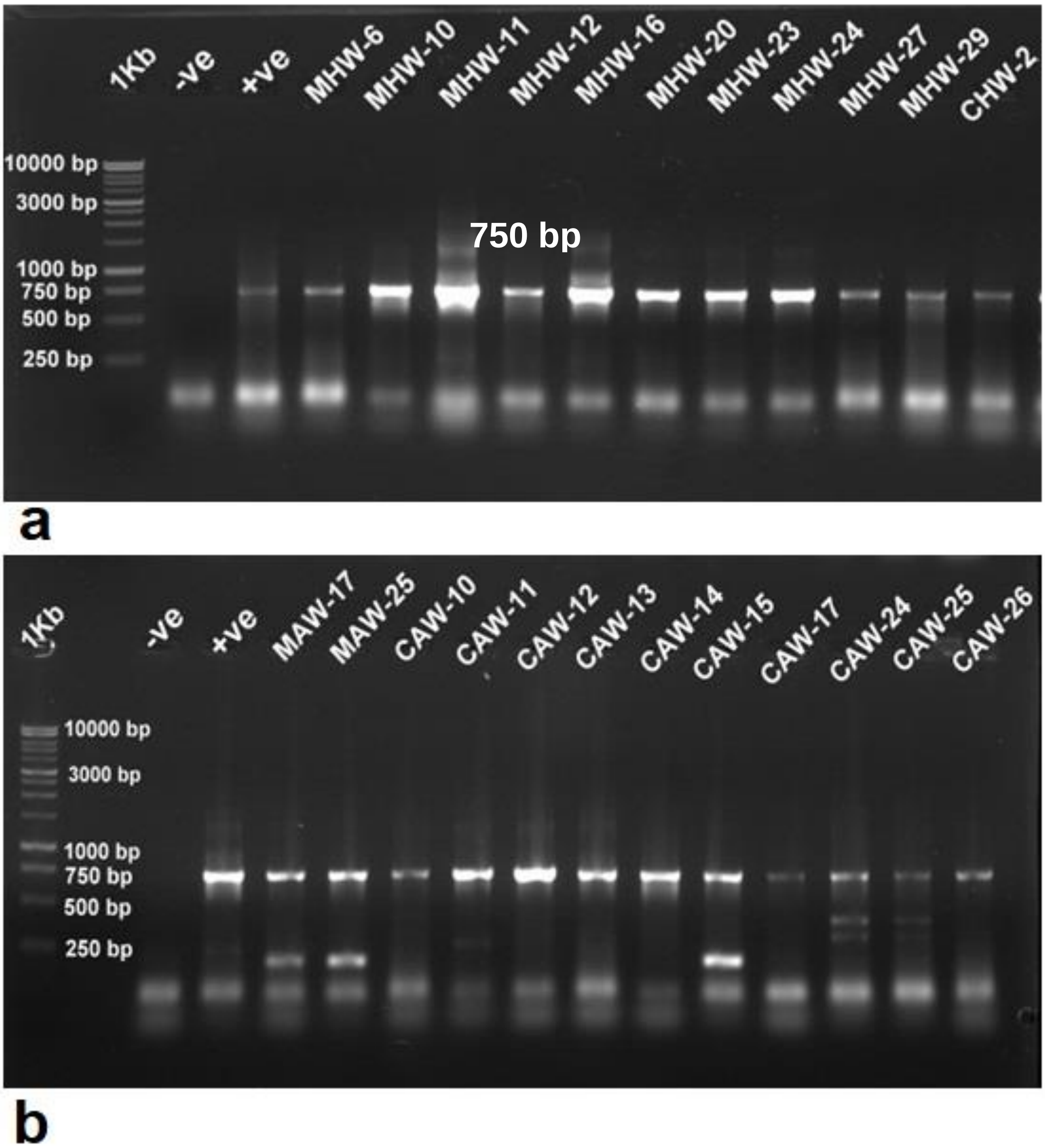

Fig. S2. Agarose gel electrophoresis (on 1% agarose gel) of PCR-specific amplicon of arsenite efflux pump gene (*ars*B) of arsenite tolerant groundwater isolates enriched from (a) heterotrophic and (b) autotrophic medium. Lane-1, 2, and 3 of (a) and (b) image indicates 1Kb (Promega, USA) DNA marker, negative control, and positive control. The other lanes of both images indicate isolate code.

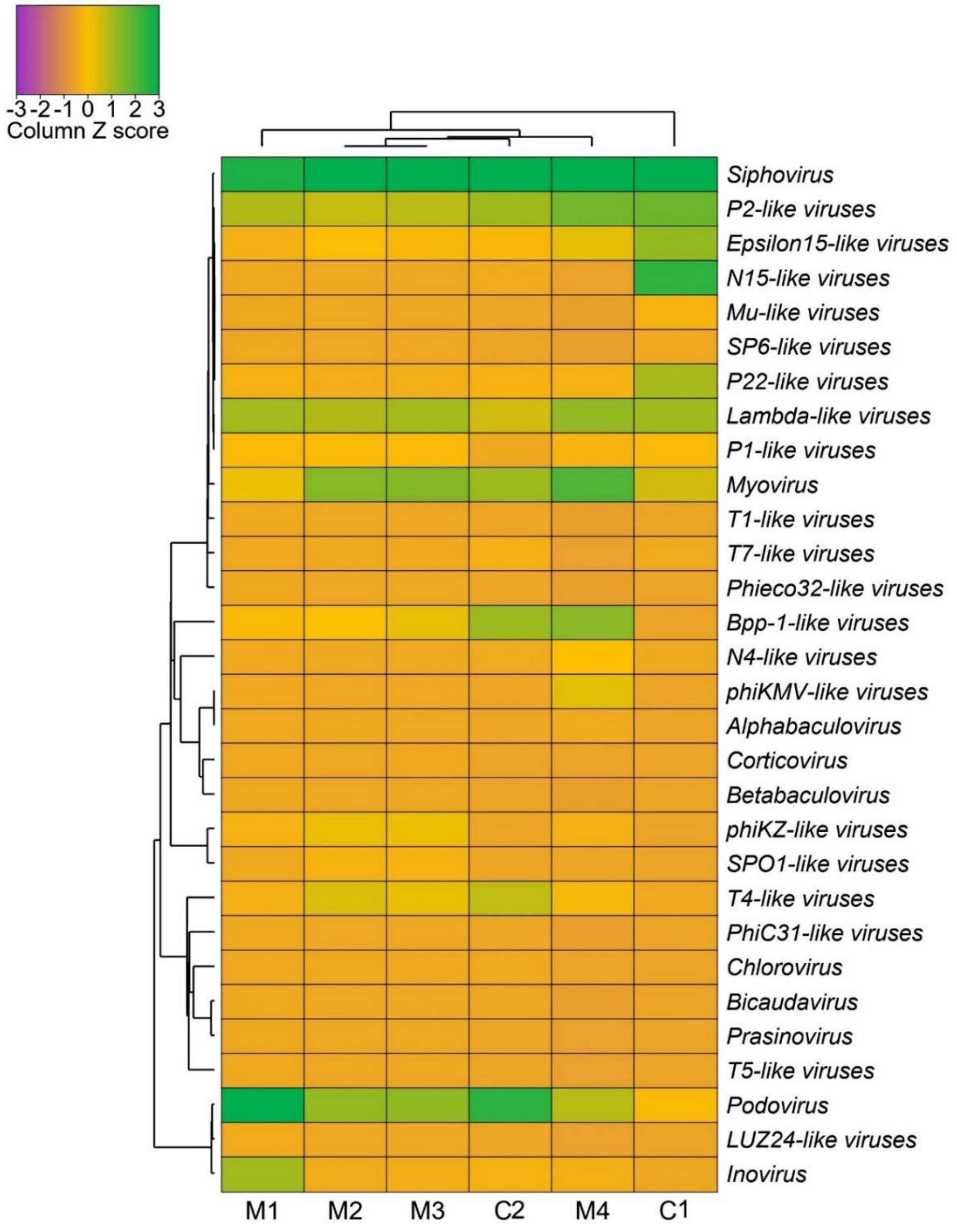

Fig. S3. The taxonomic structure of the most prevalent virus taxa in arsenic-contaminated groundwater samples. The heatmap depicts the distribution of viral genera in the Munshiganj (M1-M4) and Chandpur (C1-C2) district GW samples. The color coding reflects the presence and completeness of each viral gene, displayed as a value (Z score) ranging from -3 (low abundance) to 3 (high abundance). The green color represents the maximum abundance of the particular genes in each sample, while the purple color represents the lowest abundance.

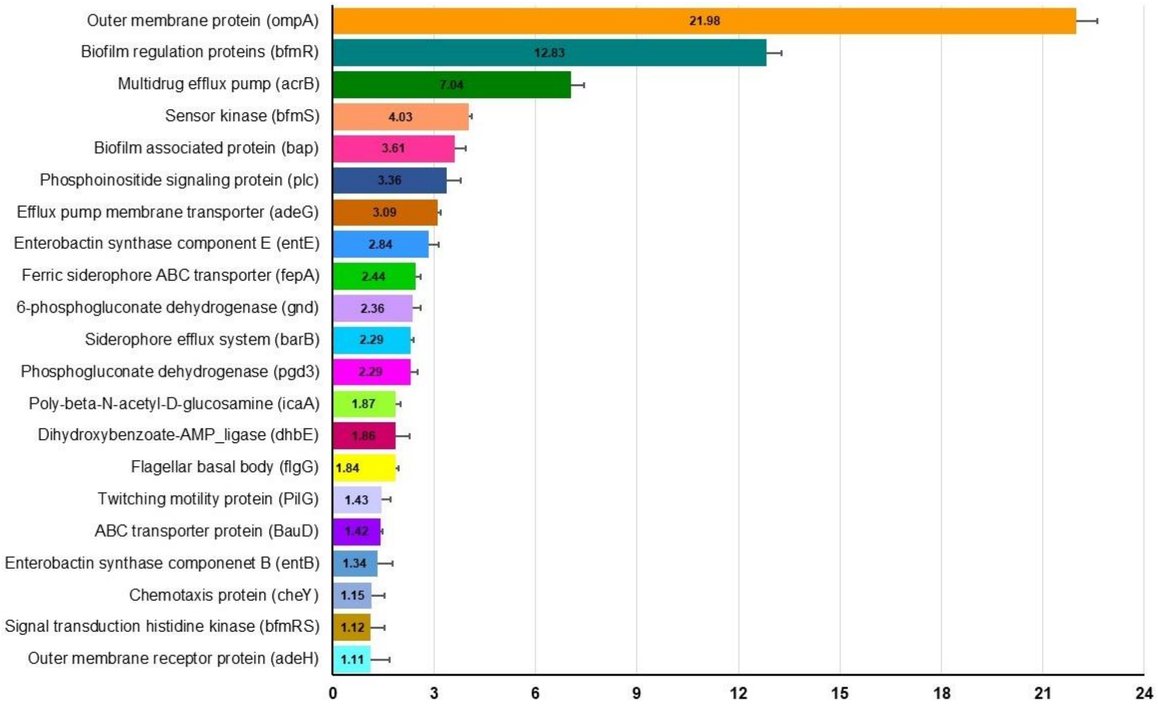

Fig. S4. Virulence factors associated genes (VFGs) detected in arsenic-contaminated GW microbiomes. The distribution of top abundant 20 VFGs found in the arsenic-polluted GW microbiomes. VFGs are represented by different colored bars according to their relative abundances. Error bars show significant differences in the relative abundances of the corresponding VFGs.

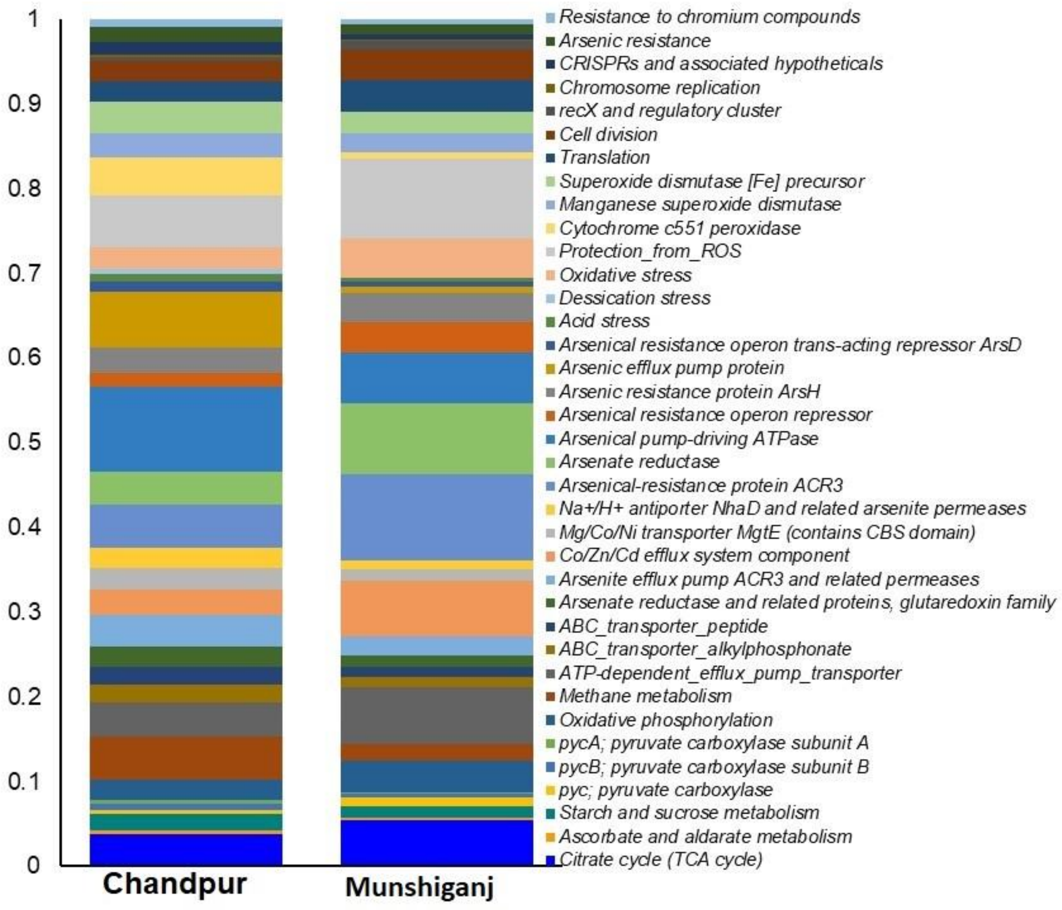

Fig. S5: Analysis of the functional genomic potentials of arsenic-contaminated GW microbial community through KEGG pathways. Between two metagenomic groups, bar charts represent the distribution of the 40 genes related to the discovered metabolic functional potentials determined using KEGG pathway analysis (Chandpur and Munshiganj). Each stacked bar plot depicts the frequency of occurrence of KEGG pathways in the relevant category's samples (Chandpur and Munshiganj district).

Table S1: Cultural and molecular features of potential arsenite resistant and oxidizing bacteria retrieved from Munshiganj and Chandpur district.

Sampling sites	Sample ID	Enrichment Type	Isolates ID	Functional markers		MIC	Phylogenetic identification
				Oxidation (*aio*A)	Resistance (*ars*B)		
**Munshiganj**	**M-1**	**Heterotrophic**	MHW-1	-	-	ND	ND
			MHW- 2	-	-	4	*Acinetobacter sp.*
			MHW-3	-	-	4	ND
			MHW-4	-	-	6	*Lysinibacillus sp.*
			MHW-5	-	-	ND	ND
	**M-2**		MHW-6	-	+	12	ND
			MHW-7	-	-	ND	ND
			MHW-8	-	-	10	*Acinetobacter sp.*
			MHW-9	-	-	ND	ND
			MHW-10,	-	+	10	ND
			MHW-11	-	+	10	*Comamonas sp.*
			MHW-12	-	+	8	ND
			MHW-13	-	-	ND	ND
			MHW-14	-	-	ND	ND
			MHW-15	-	-	ND	ND
			MHW-16	-	+	ND	ND
	**M-3**		MHW-17	-	-	ND	ND
			MHW-18	-	-	ND	ND
			MHW-19	-	-	4	*Kluyvera sp.*
			MHW- 20	-	+	8	ND
			MHW-21	-	-	ND	ND
			MHW-22	-	-	4	ND
	**M-4**		MHW-23	-	+	6	*Klebsiella sp.*
			MHW-24	-	+	12	*Pseudomonas sp.*

			MHW-25	-	-	ND	ND
			MHW-26	-	-	ND	ND
			MHW-27	-	+	15	*Stenotrophomonas sp.*
			MHW-28	-	-	12	*Stenotrophomonas sp.*
			MHW-29	-	+	10	*Comamonas sp.*
			MHW-30	-	-	ND	ND
	**M-1**	**Autotrophic**	MAW-1	-	-	ND	ND
			MAW-2	-	-	15	ND
			MAW-3	-	-	ND	ND
			MAW-4	-	-	ND	ND
			MAW-6	-	-	15	ND
	**M-2**		MAW-7	-	-	18	ND
			MAW-8	-	-	12	ND
			MAW- 9	-	-	12	ND
			MAW-10	-	-	ND	ND
			MAW-11	-	-	ND	ND
			MAW-13	-	-	ND	ND
			MAW-14	-	-	ND	ND
	**M-3**		MAW-16	-	-	ND	ND
			MAW-17	-	+	ND	ND
			MAW-18	-	-	ND	ND
			MAW-19	-	-	ND	ND
			MAW-20	-	-	ND	ND
	**M-4**		MAW-22	-	-	25	ND
			MAW-23	-	-	25	ND
			MAW-24	+	-	8	*Parburkholderia sp.*
			MAW-25	-	+	20	ND
			MAW-26	-	-	25	ND
			MAW-27	-	-	ND	ND

			MAW-28	-	-	ND	ND
**Chandpur**	**C-4**	**Heterotrophic**	CHW-1	+	-	6	*Achromobacter sp.*
			CHW-2	-	+	10	*Pseudomonas sp.*
			CHW-7	-	-	4	*Comamonas sp.*
	**C-5**		CHW-23	-	-	ND	ND
			CHW-24	-	-	ND	ND
			CHW-25,	-	-	ND	ND
			CHW-26	-	-	ND	ND
			CHW-27	-	-	6	ND
	**C-4**	**Autotrophic**	CAW-13	+	+	8	ND
			CAW-14	+	+	12	ND
			CAW-15	+	+	12	ND
			CAW-17	+	+	26	ND
			CAW-24	+	+	2	*Parburkholderia sp.*
			CAW-25	+	+	32	*Stenotrophomonas sp.*
			CAW-26	+	+	12	ND
	**C-5**		CAW-10	-	+	8	*Pseudomonas sp.*
			CAW-11	+	+	18	ND
			CAW-12	+	+	10	ND

*MHW: Munshiganj heterotrophic water; MAW: Munshiganj autotrophic water; CHW: Chandpur heterotrophic water, CAW: Chandpur autotrophic water, ND: Not determined.
